# Experimental evidence for temporal uncoupling of brain Aβ deposition and neurodegenerative sequelae

**DOI:** 10.1038/s41467-022-34538-5

**Published:** 2022-11-28

**Authors:** Christine Rother, Ruth E. Uhlmann, Stephan A. Müller, Juliane Schelle, Angelos Skodras, Ulrike Obermüller, Lisa M. Häsler, Marius Lambert, Frank Baumann, Ying Xu, Carina Bergmann, Giulia Salvadori, Maarten Loos, Irena Brzak, Derya Shimshek, Ulf Neumann, Dominantly Inherited Alzheimer Network, Lary C. Walker, Stephanie A. Schultz, Jasmeer P. Chhatwal, Stephan A. Kaeser, Stefan F. Lichtenthaler, Matthias Staufenbiel, Mathias Jucker

**Affiliations:** 1 Department of Cellular Neurology, Hertie Institute for Clinical Brain Research, University of Tübingen, D-72076 Tübingen, Germany; 2 German Center for Neurodegenerative Diseases (DZNE), Tübingen, D-72076 Tübingen, Germany; 3 Graduate School of Cellular and Molecular Neuroscience, University of Tübingen, D-72074 Tübingen, Germany; 4 German Center for Neurodegenerative Diseases (DZNE), Munich, Germany; 5 Neuroproteomics, School of Medicine, Klinikum rechts der Isar, Technical University of Munich, Munich, Germany; 6 Munich Cluster for Systems Neurology (SyNergy), Munich, Germany; 7 Sylics (Synaptologics BV), 3721 MA Bilthoven, The Netherlands; 8 Novartis Institutes for Biomedical Research, CH-4056 Basel, Switzerland; 9 Department of Neurology and Emory National Primate Research Center, Emory University, Atlanta, GA 30322 USA; 10 Department of Neurology, Massachusetts General Hospital, Boston, MA USA

**Keywords:** Diseases of the nervous system, Biomarkers

## Abstract

Brain Aβ deposition is a key early event in the pathogenesis of Alzheimer´s disease (AD), but the long presymptomatic phase and poor correlation between Aβ deposition and clinical symptoms remain puzzling. To elucidate the dependency of downstream pathologies on Aβ, we analyzed the trajectories of cerebral Aβ accumulation, Aβ seeding activity, and neurofilament light chain (NfL) in the CSF (a biomarker of neurodegeneration) in Aβ-precursor protein transgenic mice. We find that Aβ deposition increases linearly until it reaches an apparent plateau at a late age, while Aβ seeding activity increases more rapidly and reaches a plateau earlier, coinciding with the onset of a robust increase of CSF NfL. Short-term inhibition of Aβ generation in amyloid-laden mice reduced Aβ deposition and associated glial changes, but failed to reduce Aβ seeding activity, and CSF NfL continued to increase although at a slower pace. When short-term or long-term inhibition of Aβ generation was started at pre-amyloid stages, CSF NfL did not increase despite some Aβ deposition, microglial activation, and robust brain Aβ seeding activity. A dissociation of Aβ load and CSF NfL trajectories was also found in familial AD, consistent with the view that Aβ aggregation is not kinetically coupled to neurotoxicity. Rather, neurodegeneration starts when Aβ seeding activity is saturated and before Aβ deposition reaches critical (half-maximal) levels, a phenomenon reminiscent of the two pathogenic phases in prion disease.

## Abstract (web summary)

The poor correlation between brain Aβ deposition and clinical symptoms in Alzheimer´s disease remains puzzling. Here, the authors show a temporal dissociation of Aβ deposition and neurodegeneration.

## Introduction

Protein aggregation is a central feature of many neurodegenerative diseases. In these proteopathies, disease-specific proteins adopt a self-replicating, β-strand-rich oligomeric structure that can aggregate into protofibrils or mature amyloid fibrils^1^. In AD, the Aβ peptide is generated through proteolytic processing of the Aβ-precursor protein (APP), with the β-site APP-cleaving enzyme 1 (BACE1) as the rate-limiting step^2^. Self-replicating Aβ seeds and the intracerebral deposition of Aβ in the form of amyloid plaques are observed at least two decades before the clinical signs and symptoms of AD emerge^3,4^.

The amyloid-cascade hypothesis states that Aβ deposition is the key causal event in the pathogenesis of AD^5^. However, the long delay and poor correlation between Aβ deposition and clinical symptoms, along with the lack of robust clinical benefit in Aβ-targeting trials, suggest that the AD pathogenic cascade may at later stages become partially or completely independent of Aβ aggregation^6–8^. While there is consensus that Aβ-targeting clinical trials need to be initiated in the presymptomatic phase and as early as possible^8–10^, the time point at which interference with Aβ aggregation has maximal benefit is not known.

We sought to study the link between Aβ deposition and neurodegeneration in a mouse model at different stages of AD-like pathology. Here, we analyzed the deposition and seeding activity of Aβ and how this relates to biomarkers of neurodegeneration and glial activation. We then probed this linkage by impeding Aβ production at different stages of the proteopathic cascade using BACE1 inhibition. Finally, to extend these findings to humans, we compared the trajectories of key biomarkers in the mouse model to those in cases of familial AD.

## Results

### Trajectories of brain Aβ deposition, Aβ seeding activity, and CSF NfL

**Fig. 1:**
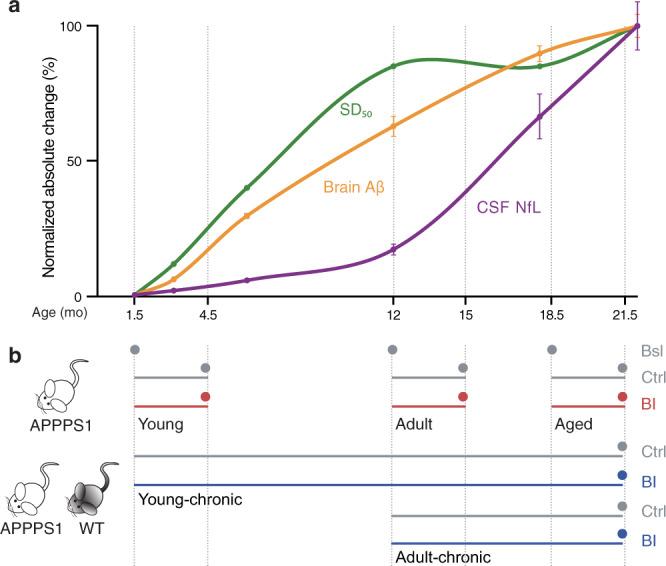
Age-dependent biomarker changes in APPPS1 mice and experimental group design. **a** Normalized absolute changes (%) in brain Aβ levels, brain Aβ seeding activity (SD_50_), and CSF NfL as a function of age in APPPS1 mice. Data were largely taken from previous publications (for brain Aβ measured by immunoassays^13^; for in vivo Aβ seeding activity (Reed-Muench method)^13^; for CSF NfL^11^ and from in-house mouse bio-/databank (see Supplementary Table 1; note that, from each of the NfL values of APPPS1 mice, the mean of the NfL values of age-matched wild-type mice was subtracted since wild-type mice also show an age-related increase^59^). Means and ± s.e.m. are shown and curves were generated in Numbers (Apple Inc., Cupertino, CA) using the “curved connection line” option. Results reveal a steady increase in brain Aβ until it slows down at a late age, whereas Aβ seeding activity increases more rapidly and reaches a plateau as early as ~12-mo of age (see also Fig. 3c where log SD_50_ data are plotted using the curve-fitting method). CSF NfL increases slowly until ~10–11-mo of age, after which a more rapid increase takes place. **b** Experimental treatment groups cover distinct time-points of decisive biomarker changes. Mice were treated either with BACE1 inhibitor- (BI) containing food pellets (red) or control (Ctrl) food pellets (gray). Three-month treatment periods (short-term) started when animals were 1.5 (young), 12 (adult), or 18.5 (aged) mo of age, and mice were analyzed at the end of each 3-month treatment period (dots). To assess baseline levels (Bsl), untreated mice were also collected at 1.5, 12, and 18.5 mo of age (dots). Chronic BI treatments (blue) started at 1.5 or 12 months of age and lasted until 21.5 months (young-chronic and adult-chronic, respectively) when the animals were sacrificed. Chronic BI treatments were also administered to control wildtype (WT) mice. Number of mice per group was *n* = 9–16; for exact numbers see Supplementary Fig. 1a.

We generated profiles of the relative age-associated changes of brain Aβ load, brain Aβ seeding activity, and NfL in the CSF of APPPS1 mice (Fig. 1a). CSF NfL is a fluid biomarker of neurodegeneration and/or neuronal dysfunction in mouse models and human cerebral proteopathies^11,12^. While the brain Aβ load increases almost linearly in APPPS1 mice until it reaches an apparent plateau at a late age (>18 mo of age), brain Aβ seeding activity increases more rapidly and reaches a plateau already in young adult mice (~12 mo of age). In contrast to both, CSF NfL shows a long lag phase with only a small increase until ~10 mo of age, after which a rapid increase begins (Fig. 1a). The last time point analyzed was 22 mo of age, which corresponds to the mean life expectancy of the APPPS1 mouse line^13^.

To study relationships and putative causalities of these changes, we then blocked Aβ generation by the administration of a BACE1 inhibitor at different disease stages (Fig. 1b). Short-term (3 months) Aβ inhibition was undertaken in young, 1.5-mo-old APPPS1 mice that do not yet show robust Aβ deposition. The same short-term Aβ inhibition protocol was performed in adult, 12-mo-old mice (an age at which Aβ deposition increases linearly and Aβ seeding activity reaches a plateau) and in aged, 18.5-mo-old mice (an age at which Aβ deposition is near maximum and seeding activity has plateaued, but NfL levels are increasing rapidly). All of these mice were sacrificed immediately following treatment. Long-term BACE1 inhibition was undertaken either from 1.5 mo to 21.5 mo of age (young-chronic) or from 12 mo to 21.5 mo (adult-chronic), after which the mice were sacrificed (Fig. 1b; for mouse numbers see Supplementary Fig. 1a).

### Stage-specific inhibition of Aβ generation

**Fig. 2:**
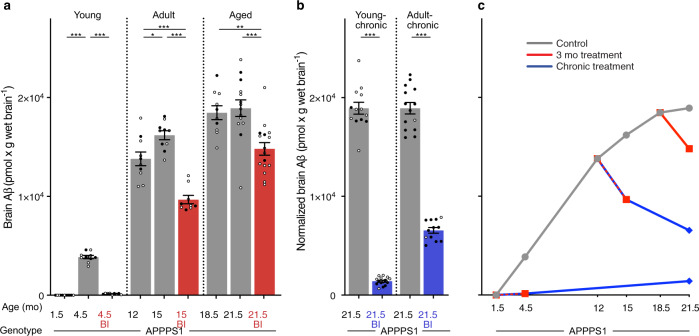
Brain Aβ after short-term and chronic BACE1 inhibition. Brain Aβ (human Aβx–40 and Aβx–42 assessed by immunoassays) in APPPS1 mice. **a** Brain Aβ at baseline and after short-term BI treatment in ‘young’, ‘adult’, and ‘aged’ mice (see Fig. 1 and Supplementary Fig. 1a for treatment groups and number of mice per group). Short-term BI treatment caused a significant decrease in brain Aβ compared to the respective age-matched control groups, and was below baseline in the ‘adult’ and ‘aged’ groups (ANOVA, ‘young’: F(2, 27) = 547.1; ‘adult’: F(2, 26) = 35.31; ‘aged’: F(2, 37) = 10.33, all *P* < 0.001; post hoc Tukey’s multiple comparisons, *P < 0.05, **P < 0.01, ***P < 0.001). **b** Brain Aβ levels in the young-chronic and adult-chronic groups were normalized to the 21.5 mo-old control mice in the 3-month treatment group shown in **a**. Two-tailed unpaired *t*-tests revealed significantly lower brain Aβ levels in the BI-treated mice (‘young-chronic’: *t*(26)=30.69; ‘adult-chronic’: *t*(24) = 17.99, both ***P < 0.001). **c** Cross-sectional curves of the means of brain Aβ from **a** and **b** show the increase of brain Aβ in the control mice (gray line), consistent with previous studies shown in Fig. 1a. When initiated before Aβ deposition was present (i.e., at 1.5 mo of age), BI treatment markedly (>90%) inhibited the deposition of Aβ for both the short-term and chronic treatments. When initiated in amyloid-laden mice, the BI treatment led to an Aβ reduction below baseline. Since the chronic treatments are, in a sense, an extension of the 3-month treatments, the lines are drawn from the 3-month treatments to the end of the chronic treatments. All data are represented as group means ± s.e.m. Open circles are males, filled circles females; no effect of sex was found (see Methods). Similar data were obtained when Aβ deposition was assessed by immunostaining, see Supplementary Fig. 2.

The BACE1 inhibitor NB-360 (BI) was used to block Aβ generation^14^. Delivered in food pellets, the BI reduces Aβ production in mice by more than 90%^14–16^. Indeed, short-term treatment of young APPPS1 mice largely (>95%) prevented brain Aβ deposition (i.e., 4.5-mo-old BI-treated mice had only 4% of total Aβ compared to control APPPS1 mice), while in 15- and 21.5-mo-old APPPS1 mice, short-term treatment not only prevented further increases, but even reduced brain Aβ load below baseline (i.e., the time point when the treatment started) (Fig. 2a). Chronic treatment was found to sustain the effect of the short-term treatments: Mice chronically treated with the BI from 1.5 to 21.5 mo of age showed only 7% of total brain Aβ compared to 21.5-mo-old untreated APPPS1 mice, while chronic treatment of adult mice from 12 to 21.5 mo of age reduced Aβ load by almost 50% (Fig. 2b, c).

Overall similar results were obtained when Aβ was assessed by immunohistochemical analysis (Supplementary Fig. 2). However, based on immunohistochemistry Aβ reduction below baseline in the 15- and 21.5-mo-old short-term-treated mice appeared to be less pronounced compared to the levels measured biochemically. Similarly, immunohistochemical analysis of Aβ after short-term and chronic, long-term BI treatment revealed 15 and 31% of the immunostaining of 4.5- and 21.5-mo-old untreated APPPS1 mice (while using biochemical measurements it was 4 and 7%, respectively, see above). These observations may indicate that Aβ deposits after BI treatment are less densely packed, or that BI treatment removes Aβ at sites (e.g., at the synapse) that evade our quantitative histological analysis (as observed in a single human case after verubecestat [another BACE1 inhibitor] treatment^17^). It is also possible that BI treatment leads to a relative increase in C-truncated Aβ species that are not captured by our current Aβx–40 and Aβx–42 immunoassays^18^.

### Changes in microglia and Tau

Microglial activation is tightly associated with Aβ deposition in mouse models of β-amyloidosis and in humans^19,20^. Indeed, when microglial activation was assessed in our various treatment groups, either by measuring brain soluble Trem2 (sTrem2^21,22^) or immunohistochemically using Iba1-staining, virtually the same results as for Aβ were achieved (Supplementary Fig. 3). Notably again, short-term BI treatment reduced microglial activation below baseline in 15- and 21.5-mo-old mice, while long-term BI treatment from 1.5 to 21.5 mo resulted in less than 20% of the levels of Iba-1 staining and sTrem2 that were found in 21.5-mo-old untreated APPPS1 mice.

An increase of CSF Tau levels (or its phosphorylated species, pTau) is also associated with cerebral Aβ deposition^23–26^. Consistently, CSF Tau generally followed the age-related increase in Aβ deposition in the untreated APPPS1 mice (Supplementary Fig. 4). However, the increase of Tau levels after the short-term BI treatment was only blocked but not reduced below baseline, whereas chronic BI treatment in the adult mice did reduce Tau levels below baseline (Supplementary Fig. 4).

### Discordance of Aβ deposition and Aβ seeding activity

**Fig. 3:**
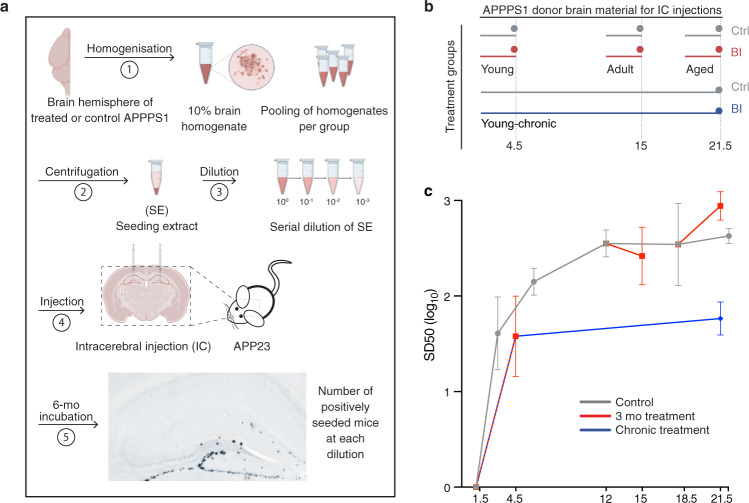
Brain Aβ seeding activity after short-term and chronic BACE1 inhibition. **a** Brain extracts from all mice within a group were pooled, serially diluted, and intracerebrally (IC) injected into the hippocampus of young, pre-depositing 2- to 3-mo old female APP23 host mice (*n* = 4–6 per extract; for exact numbers see Supplementary Fig. 1b). APP23 host mice were analyzed for Aβ deposition using immunohistochemistry (CN6 and Congo Red) 6 months later. Illustrations were partly created with BioRender.com. **b** Treatment groups for which SD_50_ was determined (short-term for young, adult, aged; young-chronic). **c** SD_50_ (defined as the log 10 of the brain extract dilution at which 50% of the host mice showed induced Aβ deposition) was computed for each treatment group and complemented with the trajectories of SD_50_ from a former study (see Supplementary Fig. 1b, c and Methods). BI treatments in amyloid-laden adult and aged mice did not consistently affect the seeding activity. When BI treatment was initiated before appreciable Aβ deposition was present (i.e., at 1.5 mo of age) SD_50_ almost reached control levels after the 3-month treatment. After chronic treatment, SD_50_ was about 1 log (10-fold dilution) below the control (i.e., below the saturated seeding activity). Since the chronic treatment is, in a sense, an extension of the 3-month treatment, the line is drawn from the 3-month treatments to the end of the chronic treatment, suggesting that the SD_50_ remains at this level when Aβ generation is continuously blocked.

To assess the brain Aβ seeding activity of BI-treated APPPS1 mice, a well-established endpoint titration in vivo seeding assay^13^ was carried out (Fig. 3). To reduce the number of experimental mice, and in line with the 3Rs (Replace, Reduce, Refine) principles, previously published brain seeding dose 50 (SD_50_) data of untreated APPPS1 mice were incorporated into the present analysis (Fig. 3).

In contrast to the changes in microglia and CSF Tau that overall followed the trajectory of Aβ deposition, brain Aβ seeding activity behaved differently. In the young APPPS1 mice, short-term BI treatment largely prevented Aβ deposition in brain (only 4–15% of that of controls, for immunoassay and histology, respectively; see above), but SD_50_ increased and nearly reached the levels of untreated APPPS1 control mice (Fig. 3c). In contrast, brain Aβ seeding activity after chronic BI treatment was about 10-fold lower (one common-log scale) compared to that of controls. Although true longitudinal analysis was not performed, both observations together suggest that, after an initial rapid increase of SD_50_, continuous and long-term BI treatment maintains the SD_50_ one log scale below the maximal seeding activity of aged control mice (Fig. 3c). Short-term BI treatment of aged amyloid-laden APPPS1 mice did not appear to change the SD_50_ (although it should be noted that the limited animal numbers and incorporation of previously collected SD_50_ numbers make a more firm conclusion difficult) (Fig. 3c). Overall, the observations confirm the non-linear relationship between Aβ seeding activity and Aβ load as also suggested by the trajectories of these measures in aging APPPS1 mice (Fig. 1a). In other words, Aβ seeding activity remains potent even in the context of reduced Aβ generation and load.

### CSF NfL continues to increase after inhibition of Aβ generation

**Fig. 4:**
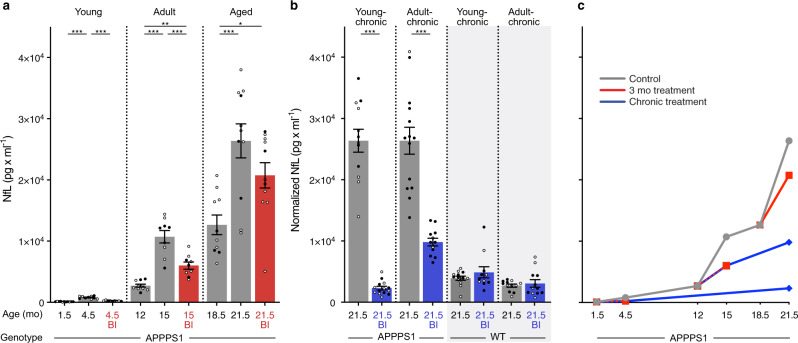
NfL in CSF after short-term and chronic BACE1 inhibition. **a** CSF NfL was measured at baseline and after short-term BI treatment in ‘young’, ‘adult’, and ‘aged’ mice (see Fig. 1 and Supplementary Fig. 1a for treatment groups and number of mice per group; note insufficient CSF amount and/or measurement errors for APPPS1 ‘adult control’, *n* = 1; ‘aged control’, *n* = 3; ‘aged BI’, *n* = 5; ‘young-chronic‘ control, *n* = 1; young-chronic’ BI, *n* = 1; and for WT ‘young-chronic’ control, *n* = 2). BI treatment in the young group prevented the NfL increase, while in the adult and aged groups, NfL still increased compared to baseline levels, albeit less than in the control groups (ANOVA, ‘young’ (F(2, 27) = 80.58; ‘adult’ F(2, 25)= 36.51; ‘aged’ F(2, 29)= 9.254, all *P* < 0.001; post hoc Tukey’s multiple comparisons, *P < 0.05, **P < 0.01, ***P < 0.001). **b** CSF NfL in the young-chronic and adult-chronic groups were normalized to the 21.5 mo-old control mice of the 3-month treatment group shown in **a**. Two-tailed unpaired *t* tests revealed significantly lower CSF NfL levels in the BI-treated APPPS1 mice (‘young chronic’: *t*(24) = 13.64; ‘adult-chronic’: *t*(24) = 6.754, bo*t*h ***P < 0.001). The same chronic treatment was also undertaken in WT mice (see Fig. 1b for treatment details), but BI treatment had no effect on CSF NfL. **c** Cross-sectional curve of the group means from **a** and **b** shows the slow initial increase of CSF NfL followed by a steep increase of NfL in adult and aged APPPS1 mice (gray line) as predicted by previous studies and shown in Fig. 1a. Although BI treatments in amyloid-laden mice slowed the NfL increase (at least in the adult group), there was still a significant increase compared to the baseline groups. Only when BI treatment was initiated before any amyloid-deposition (i.e., in the young group) could the increase of NfL be blocked. Note the similar NfL levels in chronically treated APPPS1 mice and WT mice at 21.5 months, indicating that the NfL increase in APPPS1 could in fact be completely blocked by the chronic BI treatment. Since the chronic treatments are, in a sense, an extension of the 3-month treatments, the lines are drawn from the 3-month treatments to the end of the chronic treatment. All data are represented as group means ± s.e.m. Open circles are males, filled circles females; no effect of sex was found (see Methods).

To investigate the dependency and causality between CSF NfL changes and brain Aβ, CSF NfL was assessed in all treatment groups and additionally in chronically BI-treated wildtype mice (Fig. 4). Similar to brain Aβ, all BI-treated APPPS1 groups revealed reduced CSF NfL levels compared to their respective age-matched control groups (Fig. 4). However, and in contrast to brain Aβ (and associated microglial changes), no NfL reduction below baseline was achieved, i.e., NfL still increased in the 15 mo and 21.5 mo APPPS1 mice despite reductions of brain Aβ load, microglial activation, and blockage of the increase in CSF Tau.

Strikingly, however, when BI treatment was initiated in 1.5-mo-old mice and continued until 21.5 months of age (young-chronic), the NfL increase was completely prevented (i.e., over that observed during normal aging) (Fig. 4). This observation is surprising given that these chronic, long-term BI-treated mice still exhibited Aβ deposition (with robust Aβ seeding activity, Figs. 2 and 3) and associated microglial activation (Supplementary Fig. 3). No change in NfL was found in the chronic, long-term BI-treated wildtype mice (Fig. 4b).

Confirmation of the lack of CSF NfL increase after chronic, long-term BI treatment (and thus presumably full prevention of neurodegeneration) was sought using CSF proteomic analysis (Supplementary Fig. 5; Supplementary Data 1). While myriad proteins were found to be upregulated in the aged 21.5 mo-old APPPS1 mice compared to wildtype mice (including NfL and sTrem2), long-term BI treatment largely prevented the neuronal proteomic signature seen in untreated, aged APPPS1 mice (including all three neurofilament subunits), and also—albeit less so—amyloid-associated neuroinflammatory proteins (Supplementary Fig. 5). The decreased abundance of the well-known BACE substrate Sez6 in both aged wildtype and APPPS1 mice validated the efficacy of BACE1 inhibition into old age (Supplementary Fig. 5).

### Trajectories of Aβ deposition in brain and CSF NfL in familial AD

**Fig. 5:**
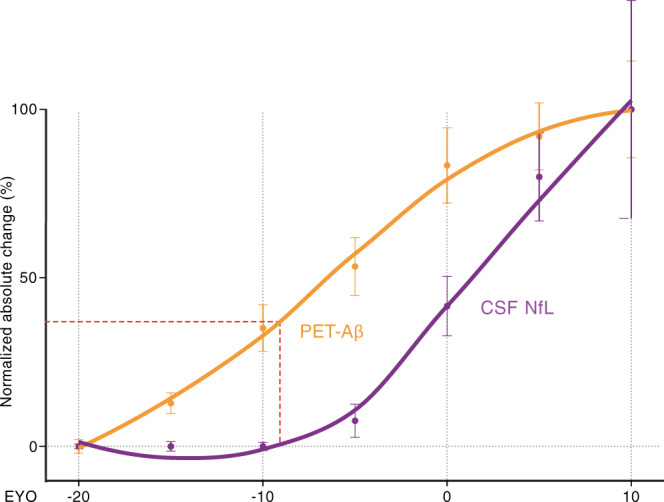
Trajectories of brain Aβ deposition and CSF NfL in familial Alzheimer´s disease. Normalized absolute changes (%) in brain Aβ-PET (using Pittsburgh Compound B; orange) and CSF NfL (purple) as a function of the estimated years to clinical symptom onset (EYO). To generate the curves, cross-sectional data from previous Dominantly Inherited Alzheimer’s Network (DIAN) publications were used (for Aβ-PET^28^; for CSF NfL^27^). For better comparison of biomarker trajectories between familial AD and the APPPS1 mouse model (see Fig. 1), Aβ-PET and CSF NfL (between EYO −20 and 10) were binned in increments of 5 years (*n* = 29, 29, 26, 31, 45, and 19 for −20 to −15, −15 to −10, −10 to −5, −5 to 0, 0 to 5, 5 to 10, respectively, for Aβ-PET; *n* = 8, 15, 13, 18, 17, and 8 for −20 to −15, −15 to −10, −10 to −5, −5 to 0, 0 to 5, 5 to 10, respectively, for CSF NfL). The relative percent change across EYO for each biomarker was calculated, and locally weighted estimated scatter plot smoothing (LOESS) was used for illustrations. Mean ± s.e.m for normalized absolute change of biomarker within each EYO bin are displayed. As in the mouse studies, the increase of CSF NfL in mutation carriers is plotted as the increase over that of non-mutation carriers, i.e., the mean NfL values of non-carrier patients were subtracted from those of the mutation carriers within each EYO bin. Of note, CSF NfL started to increase around EYO −10 when Aβ-PET reached less than half-maximal changes (red dotted line). These curves are based on cross-sectional data; if longitudinal data are used, both curves shift to lower EYO to similar degrees^27,70^.

To foster translation of our mouse work to human AD, we made use of previously published cross-sectional data from pathogenic mutation carriers in the Dominantly Inherited Alzheimer’s Network (DIAN; https://dian.wustl.edu/). DIAN participants are members of families that carry autosomal-dominant mutations that lead to AD at relatively young ages. Because the clinical onset of symptoms tends to be consistent within such families, an estimated years-to-clinical-symptom-onset (EYO) can be obtained for each participant. We used previously published data^27,28^ to visualize the relative changes in brain Aβ (using positron emission tomography [PET] with Pittsburgh Compound B) and the relative changes in neurodegeneration (again using CSF NfL) as a function of EYO (Fig. 5). Similar to brain Aβ in the APPPS1 mouse model (Fig. 1), the Aβ-PET signal in familial AD increased nearly linearly during presymptomatic disease phases, until it appears to slow down at or slightly after the time of EYO 0. In contrast, and again similar to the mouse model, CSF NfL shows a long lag phase before steeply increasing at about EYO −10 to −5. This is at least 10 years after the Aβ-PET begins to increase, and, importantly, before Aβ-PET reaches half-maximal levels (Fig. 5).

## Discussion

The link between Aβ deposition and downstream neurodegeneration in the course of AD pathogenesis is poorly understood. To study the interdependency of Aβ and neurodegeneration in the presymptomatic phase of AD, we took advantage of a well-described mouse model of cerebral β-amyloidosis that mimics the AD profile of Aβ deposition (which, in humans is indirectly assessed using Aβ-PET imaging). In both mouse model and humans, the intensity of neurodegeneration/neuronal dysfunction can be assessed by levels of NfL in the CSF^11,29^.

In APPPS1 mice, CSF NfL increased with a marked delay compared to the deposition of Aβ. Aβ deposition occurs over a period of 18 months, and the delay between the onset of Aβ deposition and a robust increase in CSF NfL is about 8–9 months (see Fig. 1). The increase in Aβ seeding activity occurred earlier and faster than did Aβ deposition, and a substantial increase of CSF NfL only started when the Aβ seeding activity had reached the plateau. This pattern is reminiscent of the two distinct pathogenic phases following prion infection in the mouse brain^30^: First there is a phase with an exponential increase in prion infectivity that rapidly reaches a plateau; this is followed by a second phase during which neurotoxicity occurs^30,31^.

To experimentally probe the dissociation of such distinct phases of cerebral β-amyloidosis, we used a BACE1 inhibitor (BI) to pharmacologically reduce the generation of Aβ at different disease stages in the APPPS1 mouse model. In amyloid-laden mice, and consistent with previous work^32^, blockage of Aβ generation led to a reduction of the Aβ load to levels below those at the start (baseline). Concomitantly, microglial activation was reduced below baseline as assessed by cellular morphology and brain sTrem2 levels (which tightly correlate with CSF sTrem2 levels^33^), and in line with the reported link between Aβ deposition and microglial activation^21^. However, despite the lowering of both Aβ load and microglial activation, CSF NfL still robustly increased in BI-treated mice (although at a slower pace compared to untreated mice). A consistent effect of the BI on the brain Aβ seeding activity was not found. Thus, at least within the parameters of our investigation, the seeding efficacy of donor brain extracts was not influenced by the level of Aβ in the host, reminiscent of the finding that the expression level of PrP has little effect on the kinetics of prion propagation^30^.

When Aβ blockage was started before any robust Aβ deposition (i.e., in 1.5 mo-old APPPS1 mice), both Aβ deposition and microglial activation reached between 10 and 30% of the levels observed in control-treated mice (for both short-term and long-term treatment). Brain Aβ seeding activity increased rapidly but then appeared to plateau at a level 10-fold below the maximal brain seeding activity of aged APPPS1 control mice. Surprisingly, however, CSF NfL did not increase above the expected age-related increase. Proteomic analysis confirmed the absence of obvious signs of neurodegeneration, but provided evidence for some glial activation. These findings are in line with the start of the CSF NfL increase coinciding roughly with the saturation of Aβ seeding activity, and an Aβ load of about half of its maximum.

Our observations in mice have salient parallels with the course of the AD presymptomatic phase of AD in humans. For familial AD, we found trajectories for brain Aβ deposition (measured by PET-Aβ) and CSF NfL to be very similar to those found in the mice, but on a different timescale. In humans as in mice, there was a substantial delay between the start of Aβ deposition and the increase in CSF NfL, i.e., at least 10 years in humans vs. 8–9 months in APPPS1 mice. Also in sporadic AD, there is a delay between the Aβ-PET increase and CSF NfL increase similar to that in familial AD^10,29,34^. Furthermore, in familial AD, CSF NfL sharply increases before half-maximal Aβ deposition is reached (Fig. 5), a pattern also reflected in the mouse model (Fig. 1). Based on these findings, it is suggested that Aβ-targeting therapies should be initiated before the half-maximal Aβ load is reached, which is roughly 5–10 years before the onset of clinical symptoms in humans. Note that the half-maximal Aβ load is a relative measure and is reached earlier with more sensitive methods of Aβ detection. Thus, if an immunohistologic/biochemical analysis could be performed in humans, it is likely that the half-maximal Aβ load would be reached earlier than with PET. In this scenario, NfL starts to increase at a somewhat higher Aβ load, a trajectory that even more closely resemble to the mouse data (Fig. 1). In either case, such a time point is much earlier than that targeted by past clinical trials that seek to reduce Aβ burden in the brain.

Indeed, BI treatment of patients with early/prodromal AD—a stage at which Aβ deposition is close to maximal—did not yield significant clinical improvement despite a modest reduction of the PET-Aβ burden^2,35,36^. A 10–15% reduction below baseline in the PET-Aβ burden was also achieved by Aβ-immunotherapy in familial AD patients (although the vast majority of patients included in the study were probably beyond half-maximal Aβ load), but cognitive deterioration still progressed and NfL increased, albeit at a slower pace^37^. The Alzheimer´s Prevention Initiative Generation Program did include asymptomatic patients, many of them at or even before half-maximal Aβ load^38^, but the study was abandoned. More recently, a lowering of Aβ deposition more than 80% below baseline has been reported in AD patients who underwent Aβ-immunotherapy^39^. Whether such drastic lowering of Aβ deposition also lowers NfL levels in humans is an exciting, if yet unexplored, possibility. Should NfL be reduced in these subjects, it can be assumed that Aβ levels above the half-maximal load still drive the NfL increase. If NfL is not lowered, it must be assumed that Aβ load past a critical threshold induces an independent pathogenic cascade that drives the increase in NfL.

Knowledge of the trajectory of Aβ seeding activity in the human AD brain is limited, but seeding activity appears to plateau at very early disease stages^40^. Also in the APPPS1 mouse model, Aβ seeding activity becomes saturated long before maximal Aβ deposition is reached. As more and more aggregates are formed, an increasing amount of newly synthesized Aβ assembles onto existing aggregates, which grow in size. Thus, the quantity of smaller aggregates, including seeds, declines^41^ yet seeds may persist for many months^42^. The increasing overall aggregation of Aβ may also deplete co-factors necessary for Aβ seeding. In the mouse models, Aβ seeding activity becomes saturated when Aβ deposition is at about half-maximal levels and CSF NfL levels begin to rise. Based on these findings, we hypothesize that the increase of CSF NfL in humans also coincides with the attainment of maximal seeding activity, reminiscent of the onset of the neurotoxic phase in prion diseases^43^.

Why the NfL increase (and thus presumed toxicity and neurodegeneration) follows Aβ accumulation and maximal seeding activity with a substantial delay, and yet continues to increase after Aβ deposition reaches a plateau, is less clear. The toxic entity may be a late-appearing Aβ species or a non-Aβ process. With increasing deposition, Aβ undergoes post-translational modifications and structural rearrangements^44,45^. It has also been suggested that new Aβ oligomers catalyzed at, or released from, the surface of Aβ deposits are the most toxic species^46–49^. Among the candidate non-Aβ species to appear later in the course of cerebral amyloidogenesis, and which promote toxicity and the pathogenesis of AD are aggregation-prone forms of Tau protein^20^. While frank neurofibrillary tangles do not develop in APP-transgenic mice, Tau accumulation in amyloid-associated dystrophic neurites does occur and soluble Tau (including pTau species) increases in the CSF in response to Aβ deposition, very similar to human (presymptomatic) AD^19,25,26,50,51^. The observation that BI treatment blocks the increase in CSF tau while NfL still increases, however, suggests that these Tau forms are likely not the toxic mediator at this stage. It is also possible that the toxic entities are more related to other aspects of neuroinflammation, lysosomal dyshomeostasis or specific cell death pathways. The observation that microglial changes after BI treatment more tightly follow the changes in Aβ deposition compared to the changes in CSF Tau (reduction below baseline vs remaining unchanged relative to baseline) suggests differences in the dependency of these markers on Aβ aggregation.

A limitation of our study is the use of mouse models overexpressing APP and PSEN1 with AD mutations, a condition that does not occur in the natural disease and thus may not recapitulate all mechanisms leading to neurodegeneration in humans. Specifically, APP-transgenic mice are models of the presymptomatic phase of AD with cerebral β-amyloidosis and early changes of tauopathy, but mice do not develop human-like neurofibrillary lesions and the symptomatic phase of AD. Nonetheless, a rapid and strong Aβ deposition seems needed in APP-transgenic mice to separate the Aβ-induced NfL increase from the normal, age-related increase CSF NfL in wildtype mice (during the short life-span of the animals).

In conclusion, our results provide experimental evidence for a temporal uncoupling of Aβ deposition and downstream neurodegenerative sequelae in a model of AD pathology. This pattern resembles the bi-phasic development of prion disease^30,31^, further substantiating the prion-like molecular properties of Aβ^52^. A dissociation of Aβ deposition and toxicity in AD has been hypothesized based on the trajectories of human biomarkers^6,7^, and is consistent with a relatively weak relationship between Aβ deposition and neurodegeneration (but a strong relationship between Aβ deposition and microgliosis) in longitudinal AD studies^27,53,54^. For clinical applications, a reliable biomarker for the time point when interference with Aβ deposition will yield maximal clinical benefit, presumably before half-maximal Aβ deposition is reached, would be of utmost importance. Biomarkers for this early phase of Aβ deposition may include novel Aβ imaging computational approaches^10^, further improvement of ultrasensitive blood Aβ (or pTau) immunoassays^55^, or fluid assays to assess brain Aβ seeding activity^56^.

## Methods

### Experimental animals

Female and male heterozygous C57BL/6J-TgN(Thy1-APPSw,Thy1-PSEN1*L166P)21 (APPPS1) mice^19^ as well as non-transgenic wildtype (WT) mice were used. Heterozygous C57BL/6J-TgN(Thy1.2-hAPP751-KM670/671NL)23 (APP23) mice^50^ were used as hosts for the in vivo seeding assay (see below). All mice were bred at the Hertie Institute for Clinical Brain Research (Tübingen, Germany) and kept under specific pathogen-free conditions. APP23 mice overexpress the human APP transgene harboring the Swedish double mutation under the neuron-specific Thy1 promoter element^50^. APPPS1 mice carry, in addition to the transgene of APP23 mice, a mutation in PSEN1, which encodes for presenilin-1, a subunit of gamma-secretase^19^. Aβ begins to deposit at 1.5 and 6 months of age in APPPS1 and APP23 mice, respectively. All animal experiments were approved by the local Animal Care and Use Committee and in accordance with the veterinary office regulations of Baden-Wuerttemberg (Germany). Pre-determination of the required sample size was done using the statistical power analysis program G*Power.

### BACE1 inhibitor treatment

APPPS1 and non-transgenic WT mice were fed either food pellets containing the BACE1 inhibitor NB-360 (Novartis) at a dose of 0.5 g NB-360/kg food pellets, or control pellets that are identical in ingredients but lacking NB-360^15,57^. Pellets were freely available to mice at any time. Such dosing yields brain exposure similar to 100μmol/kg orally^14,57^. Average plasma and brain levels of NB-360 over 24 h for this dose were 1.2 and 4.8 μM, respectively^14,57^. Distinct time-points of decisive biomarker changes were used for treatment (see Fig. 1 for experimental groups): APPPS1 mice were treated for 3 months (referred to as ‘short-term’ treatment) beginning at 1.5, 12, and 18.5 months. These mice were sacrificed at the end of the respective treatment periods, i.e., at 4.5, 15 and 21.5 mo of age. Additional groups of APPPS1 and WT mice were given long-term treatments either for most of their lives starting when young (1.5 to 21.5 months; ‘young-chronic’) or just for the later stage of their adult lives (12 to 21.5 months; ‘adult-chronic’). All long-term treated mice were sacrificed at 21.5 mo of age. Mice were randomly assigned to either NB-360 treatment or the control group (see Supplementary Fig. 1 for animal numbers and sex). Body weights of the mice were measured weekly (see Supplementary Fig. 6).

### Collection of brain and CSF

Mice received intraperitoneally an anesthetic consisting of 10% ketamine (115 mg/kg body weight) and 5% xylazine (10 mg/kg body weight) in NaCl. CSF collection was initiated when the animal showed a negative pedal reflex response as an indicator of deep pain recognition. CSF was collected as described previously^33,58^. In brief, the dura mater was punctured with a syringe (30 G, 0.3 × 8 mm needle size) to access the cisterna magna. CSF was collected using a 20 µl GELoader tip (Eppendorf Vertrieb), centrifuged at 2000 × *g* for 10 min, and frozen at −80 °C. After collection, each mouse was perfused transcardially with ice-cold PBS. The brain was removed and bisected by a mid-line sagittal cut. The lower brainstem, as well as cerebellum, were removed via a cut through the rostral midbrain. The right forebrain was fixed in 4% paraformaldehyde in PBS at 4 °C for 2 days. For cryoprotection, the forebrain was placed in 30% sucrose in PBS for 2 days and subsequently snap-frozen in 2-methyl-butane cooled by dry-ice. The left forebrain was fresh frozen on dry-ice. All brain samples were stored at −80 °C until further analyses.

### NfL measurement

The concentrations of neurofilament light chain in murine CSF (except for Fig. 1) were determined using the highly sensitive Simoa^TM^ NF-Light Advantage assay Kit (Quanterix). CSF samples were prediluted up to 1:1000 in NF-Light sample diluent and measured in duplicate on a Simoa HD-1 or HD-X Analyzer (Quanterix) according to the manufacturer’s instructions, and as reported previously^59^. Internal reference samples were measured as controls on every plate. CSF NfL levels in Fig. 1 are from a previous publication and from our intramural mouse bio- and databank, and were quantified using an electrochemiluminescence immunoassay (see legend, Fig. 1).

### Tau measurement

The concentrations of total Tau in murine CSF were determined using a murine Tau assay employing the Single Molecule Array (Simoa) technology (Quanterix) as previously described in detail^15^. CSF samples were measured at a final dilution 1:60 in duplicate on a Simoa HD-X Analyzer (Quanterix). Internal reference samples were measured as controls on every plate.

### Biochemical analysis of Aβ in brain

Brain tissue was homogenized at 10% (w/v) in sterile PBS or homogenization buffer (150 mM NaCl, 5 mM EDTA, 50 mM Tris, and Pierce protease and phosphatase inhibitor) (Thermo Fisher Scientific, Waltham, MA) with the Precellys®24 high-throughput tissue homogenizer (Bertin Technologies; 7-ml lysing tubes with 2.8-mm ceramic beads) at 5500 r.p.m. twice for 10 s with a 10 s break in between. Homogenates were aliquoted and stored at −80 °C. Aβ extraction and measurements were done as previously described^58^. Brain aliquots were thawed on ice and mixed in a ratio of 1:3.2 with ice-cold formic acid (Sigma-Aldrich; minimum purity of 96%) and sonicated on ice for 35 s. Samples were centrifuged at 25,000 × *g* at 4 °C for 1 h. The supernatant was neutralized with 1 M Tris base, 0.5 M Na_2_HPO_4_, 0.05% NaN_3_ (w/v) in a ratio of 1:20. Aβ_x-38,_ Aβ_x-40_ and Aβ_x-42_ were measured in duplicates by an electrochemiluminescence (ECL)-linked immunoassay (Meso Scale Discovery) using a commercially available V-PLEX Aβ Peptide Panel 1 (6E10) Kit. The assay was performed according to the manufacturer’s instructions. In brief, pre-coated 96-well plates were blocked for 1 h with buffer (Diluent 35) and washed three times with 0.05% Tween 20 (Carl Roth) in PBS (v/v). Formic acid extracts were diluted up to 1:300 (depending on Aβ load, to stay within the linear range of the assay) and incubated with SULFO-TAG^TM^-labeled 6E10 detection antibody for 2 h at room temperature. MSD Read Buffer T was added after washing three times (0.05% Tween 20 (Carl Roth)) in PBS (v/v) and the plate was immediately read on the Sector Imager 6000. Data analysis was performed using the MSD DISCOVERY WORKBENCH software 3.0. Internal reference samples were measured as controls on every plate. Samples measurements with a coefficient of variation (CV) > 20% of the calculated sample concentration were excluded. Because 46% of the Aβ_x-38_ measurements had a CV > 20%, only Aβ_x-40_ and Aβ_x-42_ values were used for total Aβ load. However, using a sub-analysis, no meaningful differences were observed when all Aβ_x-38_ values independent of the CV were included. For individual Aβ values that were below the assay detection limit, a fixed value (lower limit of detection of the plate divided by $\sqrt{2}$) was imputed^60^.

### Histology and immunohistochemistry

Fixed forebrains were cut coronally into 25-μm-thick sections using a freezing-sliding microtome (SM2000R; Leica Biosystems). Sections were collected in a 12-well plate and stored in cryoprotectant solution (35% ethylene glycol and 25% glycerol in PBS). Tissue sections were stained free-floating using polyclonal antibody CN6 (a follow-up antibody of CN3) directed against Aβ^61^, and microglial staining was performed with a rabbit polyclonal anti-Iba1 antibody (1:500; Wako Chemicals). Sections were mounted on slides and co-stained with Congo red according to standard protocols.

### Quantitative analysis of Aβ immunostaining

For Aβ load quantification, sets of every 12th serial, systematically sampled coronal brain section were double-stained with CN6/Congo red. Staining was performed in two batches that included either all short-term treated mice or the long-term-treated mice. On every third stained section (i.e., every 36th sampled section), stereological analysis was performed (blinded) using a microscope with a motorized XYZ stage coupled to a video microscopy system (Stereo Investigator; MBF Bioscience) as described previously^58^. The proportion of the area (%) covered by Aβ-positive staining was determined in two-dimensional sectors at a single focal plane (20 Å~/0.45 Zeiss Achroplan).

### Quantitative analysis of microglia

Quantification of the microglial activation state was performed as described previously^58^. In short, Iba1/Congo Red staining was performed on a set of every 12th coronal section for each animal. Images were acquired using a Zeiss AxioScan.Z1 slide scanner, and images were then processed using a custom-written Fiji plugin (version 2.0.0-rc-69/1.52p). Within this captured set of sections, two sections (one through the striatum and one through the dorsal hippocampus) were manually outlined and microglia were automatically identified in the selected area by using the above-mentioned script. Congo Red signal was excluded by using only the red channel of the RGB image. The image histogram was normalized to the full grayscale range. A rolling ball background subtraction filter of 100px size was used to remove the background. Objects smaller than 10 µm^2^ were excluded from the analysis, and microglia were automatically categorized into four groups based on cell size: resting (colorized red; area <50 µm^2^), resting-intermediate (colorized yellow; 50 µm^2^ ≤ area <80 µm^2^), activated (colorized green; 80 µm^2^ ≤ area <120 µm^2^) and activated, plaque-associated (colorized blue; area ≥120 µm^2^).

### Intracerebral injections and endpoint titration assay to estimate seeding dose

Brain homogenates (10% (w/v)) in sterile PBS or homogenization buffer (see biochemical analysis above) of all APPPS1 mice in each treatment group were pooled and mixed. Homogenates were centrifuged at 3000 × *g* for 5 min and the supernatant, henceforth referred to as “seeding extract”, was aliquoted and frozen at −80 °C. An aliquot was freshly thawed on ice shortly before stereotaxic injection, and the remaining extract was discarded to avoid freeze-thaw cycles. Seeding extracts were serially diluted up to 10^−4^ in sterile PBS. The dilutions necessary to calculate seeding dose 50 (SD_50_) for each treatment group was predicted a priori based on a previous publication^13^ and only these dilutions were tested. To this end 2.5 µl of the extracts were bilaterally injected into the hippocampus of 2- to 3-month-old female (randomly selected) APP23 mice (*n* = 5–7 per group, see Supplementary Fig. 1 for exact numbers). Host mice were intraperitoneally injected with 10 µl per gram bodyweight general anesthesia (fentanyl 0.05 mg/kg, midazolam 5 mg/kg, medetomidine 0.50 mg/kg). Seeding extracts were injected into the hippocampus using a Hamilton syringe (anteroposterior, −2.5 mm; left/right, ±2.0 mm; dorsoventral, −1.8 mm) at a rate of 1.25 μl/min. The syringe was held in the injection site for 2 min before being withdrawn. The incision was closed and an antidote (0.5 mg/kg flumazenil and 2.5 mg/kg atipamezole) was applied subcutaneously. Mice were constantly monitored until recovery from anesthesia.

After 6 months of incubation, APP23 host mice were sacrificed and the brains were serially sectioned into 25-µm thick coronal sections. Aβ was stained with anti-Aβ antibody CN6 and Congo Red (see histology and immunohistochemical analysis above). The SD_50_ was assessed similarly to our previously described protocol^13^. Briefly, to determine SD_50_ we computed the ratio of mice with induced Aβ deposition in the hippocampus vs. the total number of mice used for each extract dilution tested. Note that female 8- to 9-month-old APP23 mice rarely show endogenous Aβ deposition in the hippocampus. Moreover, endogenous plaques can be distinguished from induced Aβ deposition based on distribution and appearance. Only induced Aβ deposition was rated by three independent raters who were blinded to the experimental groups. Rare discordances among the raters were discussed and a common decision was achieved. The SD_50_ was then calculated according to Reed and Muench^62^ and the Spearman-Kerber Method^63,64^. Calculation with logarithmic curve-fitting was then based on the numbers Reed and Muench using Equation Log agonist vs. response with three parameters, as provided by GraphPad Prism™ version 5 and as previously described^13^.

### Quantification of soluble Trem2 in brain homogenates

Brain homogenates (10% (w/v)) were diluted 1:2 in TBS and incubated on ice for 15 min while vortexed every 5 min. Samples were ultracentrifuged at 100,000 × *g* at 4 °C for 15 min. Soluble Trem2 was measured in the supernatant using an electrochemiluminescence (ECL)-linked immunoassay (Meso Scale Discovery) as previously described^65^. Briefly, the capture antibody AF1729 (R&D Systems) was coated on a 96-well plate overnight at 4 °C. Plates were blocked with 2% bovine serum albumin in TBS with 0.5% Tween for 1 h at room temperature while being agitated (100 r.p.m.). Samples were added and incubated for 2 h at room temperature. Soluble Trem2 was decorated by the biotinylated antibody BAF1729 (R&D Systems) directed against mouse Trem2 at a concentration of 0.5 µg/ml for 1 h at room temperature. Signal detection was carried out by SULFO-TAG^TM^-labeled Streptavidin (1:400 in PBS) and MSD Read Buffer T. Between all steps, plates were washed three times with TBS containing 0.5% Tween. Plate readouts were performed immediately on a Sector Imager 600 with data analysis using MSD DISCOVERY WORKBENCH software 2.0. As controls on every plate, internal reference samples were included and recombinant mouse Trem2 (Novoprotein, Wuijang, China) from 39 pg/ml to 2500 pg/ml served as standard.

### CSF proteomics

CSF samples from 21.5-month-old APPPS1 and WT mice, with and without BACE inhibition from 1.5 to 21.5 months, were analyzed using mass spectrometry-based label-free quantification of proteins. Aliquots containing 5 µL CSF were digested with LysC and trypsin as previously described^66^. Afterwards, peptides of each sample were dissolved in 20 µL 0.1% formic acid and analyzed by mass spectrometry.

The samples were analyzed on a nanoElute LC coupled online to a timsTOF pro mass spectrometer equipped with a column toaster (Bruker, Germany). A volume of 6 µL was injected onto a 30‐cm self‐packed C18 column (75 μm ID) with 1.9 μm ReproSil‐Pur 120 C18‐AQ resin (Dr. Maisch GmbH). A binary gradient of water and acetonitrile (B) supplemented with 0.1% (v/v) formic acid was applied at a flow rate of 250 nl/min and a column temperature of 50 °C for peptide separation: 2% B 0 min; 5% B 3.5 min; 24% B 48 min; 35% B 59 min; 60% 64 min; and 85% B 65 min. A data-independent acquisition (DIA) parallel accumulation serial fragmentation (PASEF) method was used covering the m/z range from 350 to 1275 m/z with two rows of 22 m/z windows using a ramp time of 166 ms. Six steps were applied for each tims separation.

The DIA PASEF raw data were analyzed with the software DIA-NN Version 1.8^67^. First, a spectral library was generated in DIA-NN using 3 samples per experimental group (total: 12 samples) using a library-free search against a canonical protein FASTA including one sequence per gene from UniProt (download: 2021-04-09; 21998 entries). Two missed cleavages were allowed. Carbamidomethylation of cysteines was defined as static modification, whereas acetylation of protein N-termini and oxidation of methionines were defined as variable modifications. The spectral library for murine CSF contained 5949 protein groups and 37183 precursors. Afterwards, a library-based analysis was done with all samples. Protein LFQ was performed on MS2 level on the basis of at least one peptide per protein. Cross-sample normalization was disabled to account for the general protein abundance increase in APPPS1 compared to WT mouse CSF.

For statistical evaluation, the protein LFQ intensities were log2-transformed and two-sided Student’s *t* tests were used to assess group differences in each experiment. At least three quantification values per group were required for relative quantification. To account for multiple hypotheses, a permutation-based FDR threshold (*p* = 0.05; *s*_0_ = 0.1) was calculated using the software Perseus (Version 1.6.14)^68^.

### Statistical analysis

Statistics were performed using Prism 9 (GraphPad) or Microsoft Excel v.16. Data were tested for normality using the Shapiro-Wilk test. Since the majority of data passed the normality test, analyses of variance (ANOVA) were performed. Significant effects revealed by ANOVA were followed by post hoc Tukey’s multiple comparisons tests. For analyses including only two data sets, the unpaired two-sided *t* test was used to compare population means. The mean and standard error of the mean (s.e.m.) are reported for each experimental group.

Male and female mice were included in the analysis, and mice were randomly assigned to either treatment or control group (see also Supplementary Fig. 1 for animal numbers and sex). Two-way ANOVA (sex × treatment) revealed no significant sex × treatment interactions for brain Aβ, NfL or Tau for any group. For Aβ immunostaining, significant interactions for the young and adult APPPS1 groups were found. For microglia, significant interactions were found for the young, aged, and young-chronic APPPS1 groups and the adult-chronic WT group. For sTrem2, an interaction in the young-chronic group was found. However, because of the unequal numbers among some groups and the relatively small sample size per group due to subdividing the sexes, the meaning of such isolated and inconsistent findings is unclear. For the adult-chronic group, sex as a variable could not be assessed because there was only 1 and 2 males/group.

### Reporting summary

Further information on research design is available in the Nature Portfolio Reporting Summary linked to this article.

## Supplementary information

Supplementary Information

Description of Additional Supplementary Files

Supplementary Data 1

Supplementary Data 2

Reporting Summary

## Data Availability

All data supporting the findings of this study are available within the paper or in the Source Data File. APPPS1 mice are available from Papiling GmbH (support@papiling.com) and have been submitted for distribution through Jackson Laboratory (https://www.jax.org/). DIAN data are available through a request to the DIAN scientific committees and/or leadership (DIAN: dian.wustl.edu/our-research/observational-study). Mouse mass spectrometry data are provided in Supplementary Data 1 and have been deposited to the ProteomeXchange Consortium via the PRIDE partner repository^69^ with the dataset identifier PXD032782) Source data are provided with this paper.

## Source data

Source Data

## Code availability

No code was used in the course of the data acquisition or analysis.
