# Supplementary material for: Experimental evidence for temporal uncoupling of brain Aβ deposition and neurodegenerative sequelae
Source: Nat Commun. 2022 Nov 28;13:7333. doi: 10.1038/s41467-022-34538-5 (PMC9705543; doi:10.1038/s41467-022-34538-5)
Supplement: Supplementary file 1 — Supplementary Information [file 41467_2022_34538_MOESM1_ESM.pdf]

SUPPLEMENTARY INFORMATION

Experimental evidence for temporal uncoupling of brain Aβ deposition and neurodegenerative sequelae

Christine Rother[1,2,3,#], Ruth E. Uhlmann[1,2,3,#], Stephan A. Müller[4,5,6], Juliane Schelle[1,2], Angelos Skodras[1,2], Ulrike Obermüller[1,2], Lisa M. Häsler[1,2], Marius Lambert[1,2], Frank Baumann[1,2], Ying Xu[1,2], Carina Bergmann[1,2,3], Giulia Salvadori[1,2], Maarten Loos[7], Irena Brzak[8], Derya Shimshek[8], Ulf Neumann[8], Dominantly Inherited Alzheimer Network, Lary C. Walker[9], Stephanie A. Schulz[10], Jasmeer P. Chhatwal[10], Stephan A. Kaeser[1,2], Stefan F. Lichtenthaler[4,5,6], Matthias Staufenbiel[1], Mathias Jucker[1,2,*]

[1]Department of Cellular Neurology, Hertie Institute for Clinical Brain Research, University of Tübingen, D-72076 Tübingen, Germany;
[2]German Center for Neurodegenerative Diseases (DZNE), Tübingen, D-72076 Tübingen, Germany;
[3]Graduate School of Cellular and Molecular Neuroscience, University of Tübingen, D-72074 Tübingen, Germany,
[4]German Center for Neurodegenerative Diseases (DZNE), Munich, Germany
[5]Neuroproteomics, School of Medicine, Klinikum rechts der Isar, Technical University of Munich, Munich, Germany
[6]Munich Cluster for Systems Neurology (SyNergy), Munich, Germany
[7]Sylics (Synaptologics BV), 3721 MA Bilthoven, The Netherlands.
[8]Novartis Institutes for Biomedical Research, CH-4056 Basel, Switzerland.
[9]Department of Neurology and Emory National Primate Research Center, Emory University, Atlanta, GA 30322
[10]Department of Neurology, Massachusetts General Hospital, Boston, MA

Supplementary Figures 1-6

Supplementary Table 1

Member List of the Dominantly Inherited Alzheimer Network (DIAN)

a

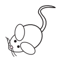 APPPS1 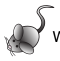 WT

	APPPS1						WT			
	Bsl		Ctrl		BI		Ctrl		BI	
	♀	♂	♀	♂	♀	♂	♀	♂	♀	♂
Young	7	4	4	6	3	6				
Adult	3	7	5	5	3	6				
Aged	3	7	3	11	3	13				
Young-chronic			4	9	7	8	6	9	7	4
Adult-chronic			13	1	10	2	6	6	7	5

b

Summary of end-point titration

Number of mice with induced Aβ deposition/ total number of mice inoculated in each group

dilution	1.2 mo	3 mo	4.5 mo	6 mo	12 mo	15 mo	18 mo	21.5 mo	22 mo
10^{0}	0/5	4/5	-/-	2/2	5/5	-/-	5/5	-/-	3/3
10^{-1}	0/5	5/5	-/-	6/6	4/4	-/-	-/-	-/-	-/-
10^{-2}	-/-	1/5	**3/5**	3/5	3/3	**5/5**	5/5	-/-	4/5
10^{-3}	-/-	-/-	-/-	0/4	0/4	-/-	0/5	**3/5**	1/3
10^{-4}	-/-	-/-	-/-	-/-	-/-	-/-	0/5	-/-	0/5
10^{0}			1/1			1/1		-/- 4/4	
10^{-1}			6/6			5/6		4/5 6/6	
10^{-2}			1/6			4/4		5/5 2/6	
10^{-3}			-/-			-/-		2/5 0/5	

c

Comparison of SD_{50} calculated for BI-treated APPPS1 mice

	young	adult	aged	young-chronic
Reed-Münch	1.60	2.38	2.83	1.75
Spearman-Kärber	1.67	2.33	2.70	1.83
Curve-fitting	1.58 ± 0.43	2.42 ± 0.3	2.94 ± 0.14	1.76 ± 0.17

Supplementary Figure 1. Number and sex of mice per group. (a) Number of APPPS1 mice and WT mice in the various groups treated with the BACE1-inhibitor (BI), control-treated (Ctrl), or analyzed at baseline (Bsl). See Fig. 1b for group description. The targeted number of mice per group was 10 for the young and adult groups and 12-15 for aged groups (i.e., short-term treatment, aged and chronic treatments). Slight deviations in numbers are the result of mouse availability from our in-house mouse colony or premature death. (Note that there was no difference in deaths between BI- and control-treated mice). Previous research has not revealed an obvious sex difference in CSF NfL or Aβ load inAPPPS1 mice[19,59], and thus both male and female mice were randomly included in the study. **(b)** Number of mice used for the endpoint titration assay to calculate Aβ seeding dose 50 of brain extracts (SD_{50}; see Fig. 3 for details). Shown are the numbers of mice with induced Aβ deposition/total number of mice inoculated per group at each brain extract dilution for the different groups. In the top rows (black) previously published data of untreated APPPS1 mice (number of mice with seeded Aβ deposition/total number of mice in each age group[13]) were incorporated into the present study in line with the 3Rs principles of reducing animal numbers. In bold, animals done in the present study. They were included for comparative analysis with the previously published numbers to make sure that the current and past inoculations results are in the same range. In the bottom rows, the short-term (3 months) BI-treated mice are shown in red while the young-chronic treated mice are shown in blue. Per group, 6-7 mice were inoculated, except for dilutions where seeding could be predicted with high confidence (two dilutions with 1 mouse only). Two groups have only 4 mice due to the death of mice. **(c)** SD_{50} calculations for the BI-treated mice in the present study using three different methods. For curve-fitting the s.e.m. is indicated and curve-fitting was used to generate the curves in Fig. 3c.

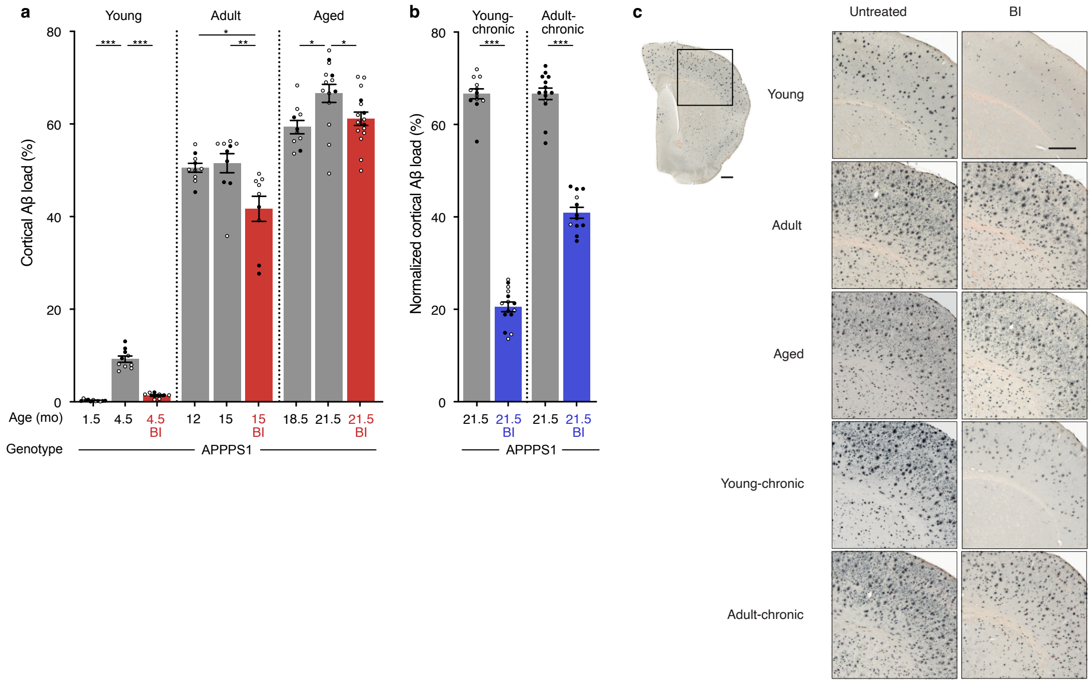

Supplementary Figure 2. Aβ load assessed by immunostaining after short-term and chronic BACE1 inhibition. The same mice were used as in Fig. 2 and outlined in Supplementary Fig. 1a. **(a)** Total Aβ immunostaining in neocortex was determined by stereological analysis, and the results mirror brain Aβ load assessed by the immunoassays shown in Fig. 2a. BI treatment caused a significant decrease in Aβ immunostaining compared to the respective age-matched control group (ANOVA, 'young': $F(2, 27)= 155.8$; 'adult': $F(2, 26)= 7.25$; 'aged': $F(2, 37)= 4.95$; all $P<0.05$; *post hoc* Tukey's multiple comparisons, $^{*}P<0.05$, $^{**}P<0.01$, $^{***}P<0.001$). **(b)** Cortical Aβ immunostaining in the young-chronic and adult-chronic groups was normalized to the 21.5 mo-old control mice of the 3-month treatment group shown in (b). Two-tailed unpaired *t*-tests show significantly lowered Aβ-immunostaining in both groups ('young chronic': $t(26)=30.99$; 'adult-chronic': $t(24)=14.77$; both $^{***}P<0.001$). All data are represented as group means ± s.e.m. Open circles are males, filled circles females; no consistent effect of sex was found (see Methods). (**c**) Representative Aβ-immunostained coronal sections (from sets of every 36th section through the neocortex) for each of the BI-treated and untreated control groups. Scale bars: 500µm.

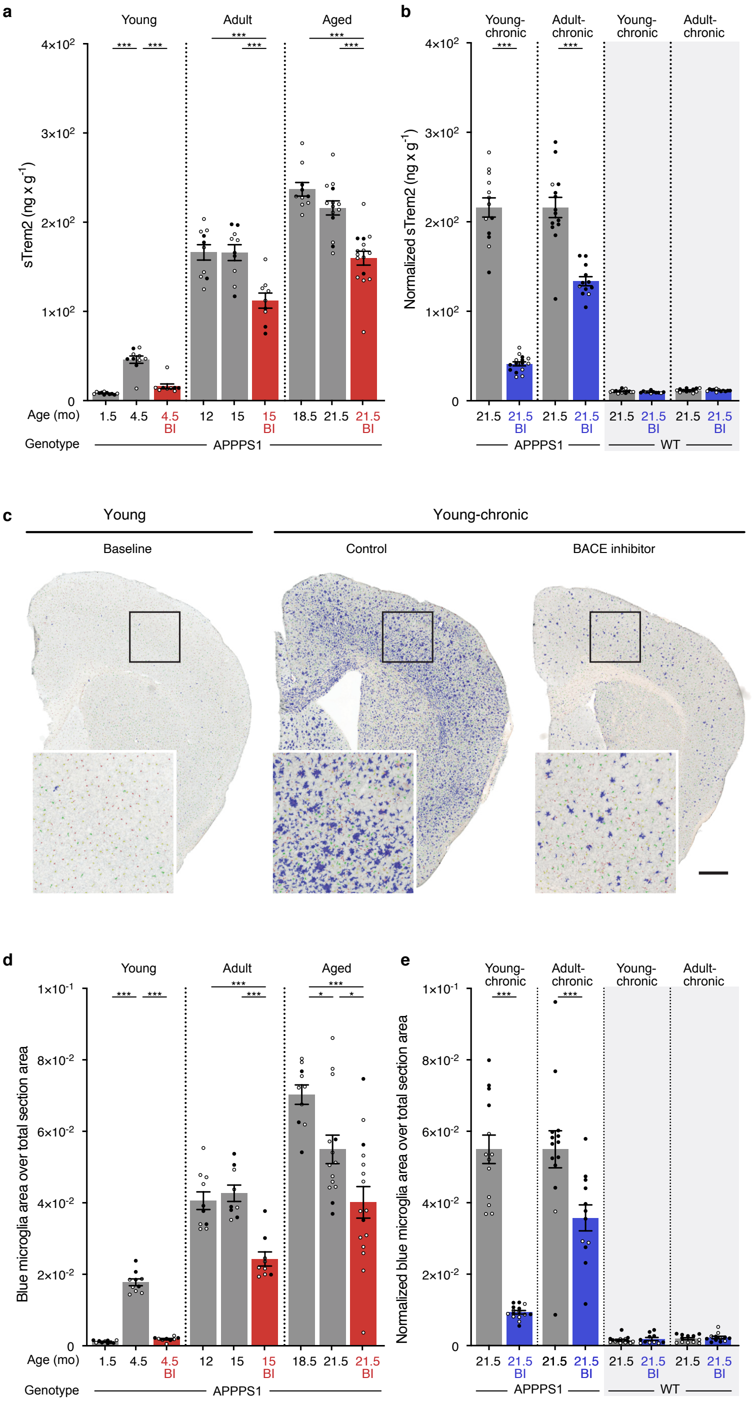

Supplementary Figure 3. Soluble Trem2 and activated microglia after short-term and chronic BACE1 inhibition. The same mice were used as in Fig. 2 and outlined in Supplementary Fig. 1a. **(a, b)** Soluble Trem2 and **(c-e)** microglial activation in brains of APPPS1 and WT mice. **(a)** BI treatment for 3 months caused a significant decrease in soluble Trem2 (sTrem2) compared to the respective age-matched control group, and sTrem2 was below baseline in the 'adult' and 'aged' groups (ANOVA, 'young' ($F(2, 27)= 54.04$; 'adult' $F(2, 26)= 12.25$; 'aged' $F(2, 37)= 25.54$, all $P<0.001$; *post hoc* Tukey's multiple comparisons, ***$P<0.001$). **(b)** Brain sTrem2 in the young-chronic and adult-chronic groups were normalized to the 21.5 mo-old control mice of the 3-month treatment group shown in (a). Two-tailed unpaired *t*-tests revealed significantly lower brain Aβ levels in the BI-treated mice ('young chronic': $t(26)=17.15$; 'adult-chronic': $t(24)=6.229$, both ***$P<0.001$). BI treatment of transgene-negative WT mice had no effect on brain sTrem2. **(c)** Iba1-immunostained microglia in the cortex of APPPS1 and WT mice

were categorized and colored based on size: Red: area <50μm^2 (resting); yellow: area 50 to < 80μm^2 (resting-intermediate); green: area 80 to <120μm^2 (activated); blue: area ≥120μm^2 (activated, plaque-associated). Representative sections from a 1.5 mo-old APPPS1 mouse (young baseline), two 21.5 mo-old APPPS1mice, one control-treated and one BI-treated mouse from 1.5 mo to 21.5 mo of age. Scale bar insert, 100μm. **(d)** Quantitative analysis revealed a significant decrease of the activated (blue) microglia over the total section area in BI-treated mice compared to controls, and additionally below baseline in the 'adult' and 'aged' groups (ANOVA, 'young' ($F(2, 26)= 279.9$; 'adult' $F(2, 25)= 18.81$; 'aged' $F(2, 37)= 12.85$, all $P<0.001$; *post hoc* Tukey's multiple comparisons, *$P<0.05$, **$P<0.01$, ***$P<0.001$). Note that one 'young baseline' and one 'adult control' mouse were excluded because of a processing error. **(e)** Activated (blue) microglia in the young-chronic and adult-chronic groups were normalized to the 21.5 mo-old control mice of the 3-month treatment group shown in (d). Two-tailed unpaired *t*-tests revealed significantly lower brain Aβ levels in the BI-treated mice ('young-chronic' and 'adult-chronic' $t(26)=12.11$; $P<0.001$ and $t(24)=2.948$; $P=0.0070$, respectively). BI treatment of WT mice had no effect on microglial activation. Note the similarities of the results of sTrem2 and activated microglia to the results for total brain Aβ (Fig. 2 and Supplementary Fig. 2). All data are represented as group means ± s.e.m. Open circles are males, filled circles females; no consistent effect of sex was found (see Methods).

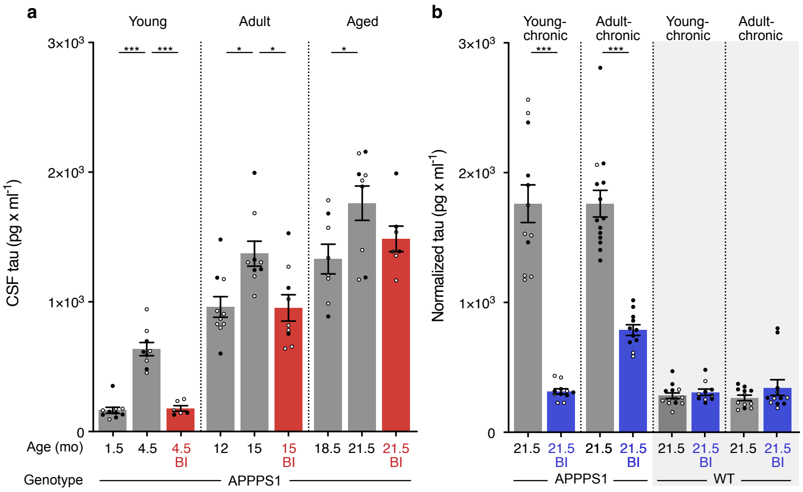

Supplementary Figure 4. CSF Tau after short-term and chronic BACE1 inhibition. CSF Tau was measured at baseline and after short-term BI treatment in 'young', 'adult', and 'aged' mice and after chronic treatments (see Fig. 1 and Supplementary Fig. 1a for treatment groups and number of mice per group – however, note that Tau measurements were performed after CSF NfL and CSF proteome measurements for mice with enough CSF remaining. Accordingly, N was 10, 9, 6 for 1,5 mo, 4.5 mo, and BI 4.5 mo; 10, 9, 9 for 12 mo, 15 mo, and BI 15 mo; 8, 9, 7 for aged 18.5 mo, 21.5 mo, and BI 21.5 mo; 12, 11 for young-chronic 21.5 mo and BI 21.5 mo; 14, 11 for adult-chronic 21.5 mo and BI 21.5 mo; 13, 10 for young-chronic WT 21.5 mo and WT BI 21.5 mo; 12, 12 for adult-chronic WT 21.5 mo and WT BI 21.5 mo). **(a)** BI treatment for 3 months completely prevented the Tau increase (note that in the aged group less than half of the CSF samples were left for analysis (ANOVA, 'young' $F(2, 22) = 56.19$; $P<0.001$; 'adult' $F(2, 25)= 6.664$, $P=0.005$; 'aged' $F(2, 21)= 3.552$, $P=0.05$; *post hoc* Tukey's multiple comparisons, $^{*}P<0.05$, $^{***}P<0.001$. **(b)** CSF Tau levels in the young-chronic and adult-chronic groups were normalized to the 21.5 mo-old control mice of the 3-month treatment group shown in (a). In the adult-chronic group, Tau levels were lowered below baseline (i.e. compared to levels at 12 mo of age) while chronic treatment fully prevented an increase in CSF Tau, given the tau levels in WT mice (two-tailed unpaired *t*-tests for 'young chronic': $t(21)=9.5$; 'adult-chronic': $t(23)=7.973$, both $^{***}P<0.001$; 'young chronic WT': $t(21)=0.7996$; 'adult-chronic WT': $t(22)=1.229$, both $P>0.05$). All data are represented as group means ± s.e.m. Open circles are males, filled circles females; no effect of sex was found (see Methods).

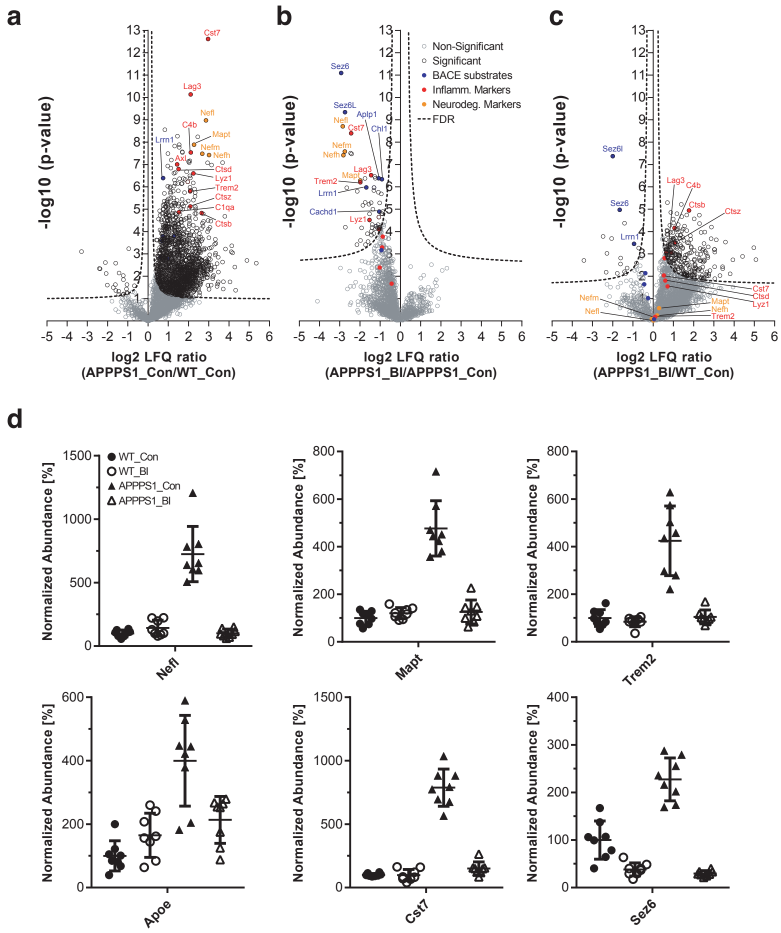

Supplementary Figure 5. CSF proteome after chronic BACE1 inhibition. The CSF of the 21.5 mo-old 'young-chronic` (1.5 to 21.5 mo) BI- and control-treated APPPS1 and wildtype (WT) mice was used for proteomic analysis (see Fig. 1 for groups; 8 animals from each group were randomly selected for this proteomic analysis). **(a)** Volcano plot comparing the CSF proteome of control-treated APPPS1 vs. control-treated WT mice (n=8 vs. n=8). Selected proteins are labeled with their UniProt gene names. Note the general increase in the abundance of many neurodegenerative markers (orange) such as Nefl, Nefm, Nefh, and Mapt (Tau) and inflammation-related proteins (red) such as Trem2, Lag3, Ctsz, and Lyz1 in APPPS1 mice compared to WT mice. **(b)** Volcano plot comparing the CSF proteome of 1.5 to 21.5 mo BI-treated vs. control-treated APPPS1 mice (n=8 vs. n=8) demonstrate that long-term BI treatment largely prevents the changes shown in (a). **(c)** Volcano plot comparing chronic (life-long) BI-treated APPPS1 mice vs. control-treated WT control mice (n=8 vs. n=8). The significant reduction of the well-known BACE1 substrates (blue) such as Sez6 and Sez6l validates the successful BACE inhibition until old age. For all Volcano plots the –log10 of the p-value of each protein is plotted against its log2 fold difference for each group comparison. The hyperbolic curves indicate the thresholds of the permutation-based FDR correction for multiple hypotheses ($p=0.05$; $s_0=0.1$). Proteins above the FDR curves (black circles) are significantly changed. **(d)** Dot plots of selected proteins. The protein LFQ intensities were normalized on the average of the WT control-treated mice (mean and SD). Note again the increase of Nefl and Mapt, and the neuroinflammatory markers sTrem2, Apoe, and Cst7 in APPPS1 mice, as well as the near complete normalization of the values by BACE inhibition. The well-known BACE substrate Sez6 shows a decreased abundance due to BACE inhibition in both WT and APPPS1 mice, validating the efficacy of BACE1 inhibition into old age. For detailed proteomic results see Supplementary Data 1.

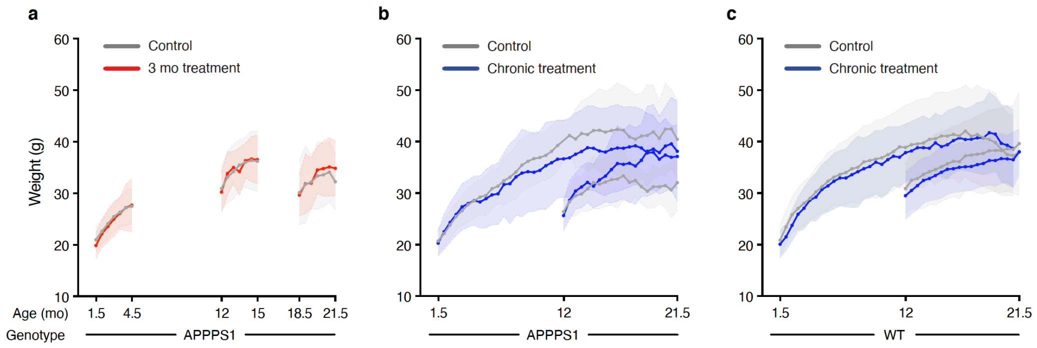

Supplementary Figure 6. Weekly body weights of BACE1-Inhibitor- and control-treated APPPS1 and wildtype mice. See Fig. 1b for group description and Supplementary Fig. 1 for mouse numbers. **(a)** No difference in body weight was observed in any of the three age groups of APPPS1 mice treated for 3 months with BI vs. control treatment. Note that, in the 'aged' 18.5 – 21.5 mo-old group, seven longitudinal datasets were incomplete. **(b, c)** Body weight of chronically treated young and adult APPPS1 mice (b) or non-transgenic WT mice (c). While BI treatment had no robust effect on body weight in the WT mice, apparent opposite changes, depending on the start of the treatment, were noted in the APPPS1 mice. Shown are group means ± SD (shaded area). Note that the BI-containing food pellets and control pellets (same pellets without the BI) were more nutritious compared to the standard lab chow, and thus animals tended to gain weight when switched to the BI-containing food pellets and control pellets.

**CSF NfL** [pg/mL] in	**Age group**					
	**1.5 mo**	**3 mo**	**6 mo**	**12 mo**	**18 mo**	**end-stage**
**APPPS1**	**63** ±3.4 (n=3)	**410** ±30.8 (n=9)	**1,255** ±85.8 (n=10)	**3,405** ±354 (n=12)	**12,475** ±1,446 (n=11)	**19,689** ±1,566 (n=9)
	61	529	1,170	4,227	5,775	23,562
	58	505	1,330	2,078	14,986	25,004
	69	318	1,491	2,727	9,559	21,252
		416	981	4,466	22,464	18,015
		510	1,631	4,718	9,573	15,134
		428	1,568	2,926	14,467	14,252
		278	1,407	2,415	10,031	14,084
		2,583	1,103	1,512	16,296	19,404
		379	1,053	3,402	6,727	26,495
		323	816	5,474	14,588	
				4,383	12,754	
				2,526		
	**1.5 mo**	**3 mo**	**6 mo**	**12 mo**	**18 mo**	**end-stage***
**WT**	**169** ±7.9 (n=3)	**239** ±27.2 (n=6)	**438** ±68.9 (n=3)	**623** ±57.4 (n=7)	**1,290** ±187.4 (n=6)	**5,903** ±1,171 (n=6)
	155	318	374	606	1,016	11,207
	171	162	575	365	926	6,566
	182	226	364	651	1,070	4,571
		190		603	974	4,935
		217		2,025	1,931	5,327
		322		888	1,825	2,814
				608		
				642		

Mean ±SEM (grey values: significant outliers according to Grubb's test; P<0.05)

* Since the mean age of the end stage WT group was 26 months compared to 22 months in the APPPS1 mice, the mean value of CSF NFL for 22-mo-old WT mice was interpolated (2,756 pg/mL) based on polynomial curve-fitting (4th degree).

Supplementary Table 1. CSF NfL values of APPPS1 mice and wildtype (WT) mice used to generate the curve in Fig. 1a.

Member List of the Dominantly Inherited Alzheimer Network (DIAN)

LastName	First	Affiliation	Email address
Adams	Sarah	Washington University in St. Louis School of Medicine	sladams@wustl.edu
Allegri	Ricardo	Institute of Neurological Research Fleni, Buenos Aires, Argentina	rallegri@fleni.org.ar
Araki	Aki	Niigata University	araki-aki@bri.niigata-u.ac.jp
Barthelemy	Nicolas	Washington University in St. Louis School of Medicine	barthelemy.nicolas@wustl.edu
Bateman	Randall	Washington University in St. Louis School of Medicine	batemanr@wustl.edu
Bechara	Jacob	Neuroscience Research Australia	j.bechara@neura.edu.au
Benzinger	Tammie	Washington University in St. Louis School of Medicine	benzingert@wustl.edu
Berman	Sarah	University of Pittsburgh	bermans@upmc.edu
Bodge	Courtney	Brown University-Butler Hospital	Cbodge@Butler.org
Brandon	Susan	Washington University in St. Louis School of Medicine	brandons@wustl.edu
Brooks	William (Bill)	Neuroscience Research Australia	w.brooks@NeuRA.edu.au
Brosch	Jared	Indiana University	
Buck	Jill	Indiana University	jilmbuck@iu.edu
Buckles	Virginia	Washington University in St. Louis School of Medicine	bucklesv@wustl.edu
Carter	Kathleen	Emory University School of Medicine	emma.kathleen.carter@emory.edu
Cash	Lisa	Washington University in St. Louis School of Medicine	cashl@wustl.edu
Chen	Charlie	Washington University in St. Louis School of Medicine	chenc@wustl.edu
Chhatwal	Jasmeer	Brigham and Women’s Hospital–Massachusetts General Hospital	Chhatwal.Jasmeer@mgh.harvard.edu
Chrem	Patricio	Institute of Neurological Research Fleni, Buenos Aires, Argentina	pchremmendez@fleni.org.ar
Chua	Jasmin	Washington University in St. Louis School of Medicine	chuajk@wustl.edu
Chui	Helena	University of Southern California	helena.chui@med.usc.edu
Cruchaga	Carlos	Washington University in St. Louis School of Medicine	cruchagac@wustl.edu
Day	Gregory S	Mayo Clinic Jacksonville	Day.gregory@mayo.edu
De La Cruz	Chrismary	Columbia University	
Denner	Darcy	Washington University in St. Louis School of Medicine	drdenner@wustl.edu
Diffenbacher	Anna	German Center for Neurodegenerative Diseases (DZNE) Munich	Anna.dieffenbacher@dzne.de
Dincer	Aylin	Washington University in St. Louis School of Medicine	aylin.dincer@wustl.edu
Donahue	Tamara	Washington University in St. Louis School of Medicine	tammie@wustl.edu
Douglas	Jane	University College London	jane.douglas@ucl.ac.uk
Duong	Duc	Emory University School of Medicine	dduong@emory.edu
Egido	Noelia	Institute of Neurological Research Fleni, Buenos Aires, Argentina	negido@fleni.org.ar
Esposito	Bianca	Icahn School of Medicine at Mount Sinai	bianca.esposito@mssm.edu
Fagan	Anne	Washington University in St. Louis School of Medicine	fanana@wustl.edu
Farlow	Marty	Indiana University	mfarlow@iupui.edu
Feldman	Becca	Washington University in St. Louis School of Medicine	rebeccaf@wustl.edu
Fitzpatrick	Colleen	Brigham and Women's Hospital-Massachusetts	cdfitzpatrick@bwh.harvard.edu
Flores	Shaney	Washington University in St. Louis School of Medicine	sflores@wustl.edu
Fox	Nick	University College London	n.fox@ucl.ac.uk
Franklin	Erin	Washington University in St. Louis School of Medicine	efranklin@wustl.edu
Friedrichsen	Nelly	Washington University in St. Louis School of Medicine	n.joseph@wustl.edu
Fujii	Hisako	Osaka City University	hfujii@med.osaka-cu.ac.jp
Gardener	Samantha	Edith Cowan University, Perth	s.gardener@ecu.edu.au
Ghetti	Bernardino	Indiana University	bghetti@iupui.edu
Goate	Alison	Icahn School of Medicine at Mount Sinai	alison.goate@mssm.edu
Goldberg	Sarah	University of Pittsburgh	goldbergs2@upmc.edu
Goldman	Jill	Columbia University	JG2673@cumc.columbia.edu
Gonzalez	Alyssa	Washington University in St. Louis School of Medicine	alyssa.gonzales@wustl.edu
Gordon	Brian	Washington University in St. Louis School of Medicine	bagordon@wustl.edu
Gräber-Sultan	Susanne	DZNE-Tübingen	susanne.graeber-sultan@dzne.de @dzne.de
Graff-Radford	Neill	Mayo Clinic Jacksonville	graffradford.neill@mayo.edu
Graham	Morgan	Mayo Clinic Jacksonville	Graham.Morgan@mayo.edu
Gray	Julia	Washington University in St. Louis School of Medicine	gray@wustl.edu
Gremminger	Emily	Washington University in St. Louis School of Medicine	egremminger@wustl.edu
Grilo	Miguel	University College London	m.grilo@ucl.ac.uk
Groves	Alex	Washington University in St. Louis School of Medicine	amgroves@wustl.edu
Haass	Christian	Ludwig-Maximilians University - Munich	Christian.Haass@mail03.med.uni-muenchen.de
Häsler	Lisa	German Center for Neurodegenerative Diseases (DZNE), Tübingen	Lisa.Haesler@dzne.de
Hassenstab	Jason	Washington University in St. Louis School of Medicine	hassenstabj@wustl.edu
Hellm	Cortaiga	Washington University in St. Louis School of Medicine	cortaiga.hellm@wustl.edu
Herries	Elizabeth	Washington University in St. Louis School of Medicine	e.herries@wustl.edu
Hoechst-Swisher	Laura	Washington University in St. Louis School of Medicine	goodl@wustl.edu
Hofmann	Anna	German Center for Neurodegenerative Diseases (DZNE), Tübingen	Anna.Hofmann@med.uni-tuebingen.de
Holtzman	David	Washington University in St. Louis School of Medicine	holtzman@wustl.edu
Hornbeck	Russ	Washington University in St. Louis School of Medicine	russ@wustl.edu
Igor	Yakushev	German Center for Neurodegnerative Diseases (DZNE) Munich	Igor.yakushev@tum.de
Ihara	Ryoko	Tokyo University	ihara-tky@umin.ac.jp
Ikeuchi	Takeshi	Niigata University	ikeuchi@bri.niigata-u.ac.jp
Ikonomovic	Snezana	University of Pittsburgh	ikonomovics@upmc.edu
Ishii	Kenji	Niigata University/Tokyo University	ishii@pet.tmig.or.jp
Jack	Clifford	Mayo Clinic Rochester	jack.clifford@mayo.edu
Jerome	Gina	Washington University in St. Louis School of Medicine	ginajerome@wustl.edu
Johnson	Erik	Emory University School of Medicine	erik.johnson@emory.edu
Jucker	Mathias	German Center for Neurodegenerative Diseases (DZNE), Tübingen	mathias.jucker@uni-tuebingen.de
Karch	Celeste	Washington University in St. Louis School of Medicine	karchc@wustl.edu
Käser	Stephan	German Center for Neurodegenerative Diseases (DZNE), Tübingen	Stephan.kaeser@uni-tuebingen.de
Kasuga	Kensaku	Niigata University	ken39@bri.niigata-u.ac.jp
Keefe	Sarah	Washington University in St. Louis School of Medicine	sarahkeefe@wustl.edu
Klunk	William (Bill)	University of Pittsburgh	klunkwe@gmail.com

Koeppe	Robert	University of Michigan	koeppe@umich.edu
Koudelis	Deb	Washington University in St. Louis School of Medicine	delanod@wustl.edu
Kuder-Buletta	Elke	German Center for Neurodegenerative Diseases (DZNE), Tübingen	elke.kuder-buletta@dzne.de
Laske	Christoph	German Center for Neurodegenerative Diseases (DZNE), Tübingen	christoph.laske@med.uni-tuebingen.de
Levey	Allan	Emory University School of Medicine	alevey@emory.edu
Levin	Johannes	German Center for Neurodegenerative Diseases (DZNE) Munich	Johannes.Levin@med.uni-muenchen.de
Li	Yan	Washington University in St. Louis School of Medicine	yanli833@wustl.edu
Lopez	Oscar	University of Pittsburgh	lopezol@upmc.edu
Marsh	Jacob	Washington University in St. Louis School of Medicine	jacobmarsh@wustl.edu
Martinez	Rita	Washington University in St. Louis School of Medicine	Ritamartinez@wustl.edu
Martins	Ralph	Edith Cowan University	r.martins@ecu.edu.au
Mason	Neal Scott	University of Pittsburgh Medical Center	masonss@upmc.edu
Masters	Colin	University of Melbourne	c.masters@unimelb.edu.au
Mawuenyega	Kwasi	Washington University in St. Louis School of Medicine	mawuenyegak@wustl.edu
McCullough	Austin	Washington University in St. Louis School of Medicine	amccullough@wustl.edu
McDade	Eric	Washington University in St. Louis School of Medicine	ericmcdade@wustl.edu
Mejia	Arlene	Columbia University	am4717@cumc.columbia.edu
Morenas-Rodriguez	Estrella	Ludwig-Maximilians University, Munich	Estrella.Morenas-Rodriguez@dzne.d
Morris	John	Washington University in St. Louis School of Medicine	jcmorris@wustl.edu
MountzMD	James	University of Pittsburgh	mountzjm@upmc.edu
Mummery	Cath	University College London	c.mummery@ucl.ac.uk
Nadkarni	Neelesh	University of Pittsburgh	nadkarnink@upmc.edu
Nagamatsu	Akemi	Tokyo University	mail:akm77-tky@umin.ac.jp
Neimeyer	Katie	Columbia University	kn2416@cumc.columbia.edu
Niimi	Yoshiki	Tokyo University	niimiy-crc@h.u-tokyo.ac.jp
Noble	James	Columbia University	jn2054@columbia.edu
Norton	Joanne	Washington University in St. Louis School of Medicine	nortonj@wustl.edu
Nuscher	Brigitte	Ludwig-Maximilians University, Munich	Brigitte.Nuscher@mail03.med.uni-muenchen.de
O'Connor	Antoinette	University College London	antoinette.o'connor@ucl.ac.uk
Obermüller	Ulricke	Hertie Institute for Clinical Brain Research	ulrike.obermueller@klinikum.uni-tuebingen.de
Patira	Riddhi	University of Pittsburgh	patirar@upmc.edu
Perrin	Richard	Washington University in St. Louis School of Medicine	rperrin@wustl.edu
Ping	Lingyan	Emory University School of Medicine	lingyan.ping@emory.edu
Preische	Oliver	German Center for Neurodegenerative Diseases (DZNE), Tübingen	Oliver.Preische@med.uni-tuebingen.de
Renton	Alan	Icahn School of Medicine at Mount Sinai	alan.renton@mssm.edu
Ringman	John	University of Southern California	john.ringman@med.usc.edu
Salloway	Stephen	Brown University-Butler Hospital	SSalloway@Butler.org
Schofield	Peter	Neuroscience Research Australia	p.schofield@neura.edu.au
Senda	Michio	Osaka City University	michio_senda@kcho.jp
Seyfried	Nick	Emory University School of Medicine	nseyfri@emory.edu
Shady	Kristine	Washington University in St. Louis School of Medicine	kesh238@g.uky.edu
Shimada	Hiroyuki	Osaka City University	h.shimada@med.osaka-cu.ac.jp
Sigurdson	Wendy	Washington University in St. Louis School of Medicine	sigurdsonw@wustl.edu
Smith	Jennifer	Washington University in St. Louis School of Medicine	smith.jennifer@wustl.edu
Smith	Lori	University of Pittsburgh	macedonials@upmc.edu
Snitz	Beth	University of Pittsburgh	snitbe@upmc.edu
Sohrabi	Hamid	Edith Cowan University	h.sohrabi@ecu.edu.au
Stephens	Sochenda	Mayo Clinic Jacksonville	Stephens.Sochenda@mayo.edu
Taddei	Kevin	Edith Cowan University	k.taddei@ecu.edu.au
Thompson	Sarah	University of Pittsburgh	thompsons24@upmc.edu
Vöglein	Jonathan	German Center for Neurodegnerative Diseases (DZNE) Munich	Jonathan.voeglein@med.uni-muenchen.de
Wang	Peter	Washington University in St. Louis School of Medicine	guoqiao@wustl.edu
Wang	Qing	Washington University in St. Louis School of Medicine	wangqing@wustl.edu
Weamer	Elise	University of Pittsburgh	weamerea@upmc.edu
Xiong	Chengjie	Washington University in St. Louis School of Medicine	chengjie@wustl.edu
Xu	Jinbin	Washington University in St. Louis School of Medicine	jinbinxu@wustl.edu
Xu	Xiong	Washington University in St. Louis School of Medicine	xxu@wustl.edu
