# Supplementary material for: Experimental evidence for temporal uncoupling of brain Aβ deposition and neurodegenerative sequelae
Source: Nat Commun. 2022 Nov 28;13:7333. doi: 10.1038/s41467-022-34538-5 (PMC9705543; doi:10.1038/s41467-022-34538-5)
Supplement: Supplementary file 2 — Description of Additional Supplementary Files [file 41467_2022_34538_MOESM2_ESM.pdf]

Description of Additional Supplementary Files

Supplementary Data 1 and 2:

Protein label-free quantification of CSF samples from 21.5 months old APPPS1and WT mice with and without BACE inhibition (for mouse IDs see Supplementary Data 1; for Mass-Spec results see Supplementary Data 2). The protein LFQ intensities were log2 transformed. A one-way ANOVA was applied to evaluate significant differences between the experimental groups. Afterwards, two-sided T-tests were applied between the individual groups. A permutation based FDR estimation (p=0.05; s0=0.1) was applied to correct for multiple hypotheses.

Source File

Reporting Summary
