# Supplementary material for: Experimental evidence for temporal uncoupling of brain Aβ deposition and neurodegenerative sequelae
Source: Nat Commun. 2022 Nov 28;13:7333. doi: 10.1038/s41467-022-34538-5 (PMC9705543; doi:10.1038/s41467-022-34538-5)
Supplement: Supplementary file 3 — Supplementary Data 1 [file 41467_2022_34538_MOESM3_ESM.docx]

**Supplementary Data 1**

ID of 21.5 months old APPPS1 and WT mice used for the protein label-free quantification of CSF samples (see Supplementary Data 2). In Supplementary Data 2 the protein LFQ intensities were log2 transformed. A one-way ANOVA was applied to evaluate significant differences between the experimental groups. Afterwards, two-sided T-tests were applied between the individual groups. A permutation based FDR estimation (p=0.05; s0=0.1) was applied to correct for multiple hypotheses.

**Group: long-term APPPS1 Group: long-term WT**

| **Mouse ID 42#** | **Mass Spec ID** | **Genotype, pos/neg** | **Sex** | **Treatment** | **Age at prep.** |
| --- | --- | --- | --- | --- | --- |
| 13572 | APP_CTL_13572 | APPPS1 pos | f | Control-Pellets | 21.5 mo |
| 13600 | APP_CTL_13600 | APPPS1 pos | m | Control-Pellets | 21.5 mo |
| 13601 | APP_CTL_13601 | APPPS1 pos | m | Control-Pellets | 21.5 mo |
| 13602 | APP_CTL_13602 | APPPS1 pos | m | Control-Pellets | 21.5 mo |
| 13603 | APP_CTL_13603 | APPPS1 pos | m | Control-Pellets | 21.5 mo |
| 13632 | APP_CTL_13632 | APPPS1 pos | m | Control-Pellets | 21.5 mo |
| 13633 | APP_CTL_13633 | APPPS1 pos | m | Control-Pellets | 21.5 mo |
| 13634 | APP_CTL_13634 | APPPS1 pos | m | Control-Pellets | 21.5 mo |
| 13559 | APP_BACE_13559 | APPPS1 pos | f | BACE-Inhibitor treated | 21.5 mo |
| 13563 | APP_BACE_13563 | APPPS1 pos | m | BACE-Inhibitor treated | 21.5 mo |
| 13566 | APP_BACE_13566 | APPPS1 pos | m | BACE-Inhibitor treated | 21.5 mo |
| 13573 | APP_BACE_13573 | APPPS1 pos | m | BACE-Inhibitor treated | 21.5 mo |
| 13576 | APP_BACE_13576 | APPPS1 pos | m | BACE-Inhibitor treated | 21.5 mo |
| 13588 | APP_BACE_13588 | APPPS1 pos | f | BACE-Inhibitor treated | 21.5 mo |
| 13611 | APP_BACE_13611 | APPPS1 pos | m | BACE-Inhibitor treated | 21.5 mo |
| 13620 | APP_BACE_13620 | APPPS1 pos | f | BACE-Inhibitor treated | 21.5 mo |

**Group: long-term WT Group: long-term WT**

| **Mouse ID 42#** |  | **Genotype, pos/neg** | **Sex** | **Treatment** | **Age at prep.** |
| --- | --- | --- | --- | --- | --- |
| 17867 | WT_CTL_17867 | APPPS1 neg | m | Control-Pellets | 21.5 mo |
| 17870 | WT_CTL_17870 | APPPS1 neg | m | Control-Pellets | 21.5 mo |
| 17888 | WT_CTL_17888 | APPPS1 neg | m | Control-Pellets | 21.5 mo |
| 17894 | WT_CTL_17894 | APPPS1 neg | m | Control-Pellets | 21.5 mo |
| 17912 | WT_CTL_17912 | APPPS1 neg | m | Control-Pellets | 21.5 mo |
| 17920 | WT_CTL_17920 | APPPS1 neg | f | Control-Pellets | 21.5 mo |
| 17931 | WT_CTL_17931 | APPPS1 neg | f | Control-Pellets | 21.5 mo |
| 17933 | WT_CTL_17933 | APPPS1 neg | f | Control-Pellets | 21.5 mo |
| 17866 | WT_BACE_17866 | APPPS1 neg | f | BACE-Inhibitor treated | 21.5 mo |
| 17876 | WT_BACE_17876 | APPPS1 neg | m | BACE-Inhibitor treated | 21.5 mo |
| 17883 | WT_BACE_17883 | APPPS1 neg | f | BACE-Inhibitor treated | 21.5 mo |
| 17889 | WT_BACE_17889 | APPPS1 neg | f | BACE-Inhibitor treated | 21.5 mo |
| 17893 | WT_BACE_17893 | APPPS1 neg | f | BACE-Inhibitor treated | 21.5 mo |
| 17903 | WT_BACE_17903 | APPPS1 neg | m | BACE-Inhibitor treated | 21.5 mo |
| 17909 | WT_BACE_17909 | APPPS1 neg | f | BACE-Inhibitor treated | 21.5 mo |
| 17942 | WT_BACE_17942 | APPPS1 neg | m | BACE-Inhibitor treated | 21.5 mo |
