# Supplementary material for: Experimental evidence for temporal uncoupling of brain Aβ deposition and neurodegenerative sequelae
Source: Nat Commun. 2022 Nov 28;13:7333. doi: 10.1038/s41467-022-34538-5 (PMC9705543; doi:10.1038/s41467-022-34538-5)
Supplement: Supplementary file 5 — Reporting Summary [file 41467_2022_34538_MOESM5_ESM.pdf]

nature portfolio

Corresponding author(s): Mathias Jucker

Last updated by author(s): Oct 15, 2022

Reporting Summary

Nature Portfolio wishes to improve the reproducibility of the work that we publish. This form provides structure for consistency and transparency in reporting. For further information on Nature Portfolio policies, see our Editorial Policies and the Editorial Policy Checklist.

Statistics

For all statistical analyses, confirm that the following items are present in the figure legend, table legend, main text, or Methods section.

n/a	Confirmed	
☐	☒	The exact sample size (n) for each experimental group/condition, given as a discrete number and unit of measurement
☐	☒	A statement on whether measurements were taken from distinct samples or whether the same sample was measured repeatedly
☐	☒	The statistical test(s) used AND whether they are one- or two-sided<br>*Only common tests should be described solely by name; describe more complex techniques in the Methods section.*
☒	☐	A description of all covariates tested
☐	☒	A description of any assumptions or corrections, such as tests of normality and adjustment for multiple comparisons
☐	☒	A full description of the statistical parameters including central tendency (e.g. means) or other basic estimates (e.g. regression coefficient) AND variation (e.g. standard deviation) or associated estimates of uncertainty (e.g. confidence intervals)
☐	☒	For null hypothesis testing, the test statistic (e.g. F, t, r) with confidence intervals, effect sizes, degrees of freedom and P value noted<br>*Give P values as exact values whenever suitable.*
☒	☐	For Bayesian analysis, information on the choice of priors and Markov chain Monte Carlo settings
☒	☐	For hierarchical and complex designs, identification of the appropriate level for tests and full reporting of outcomes
☒	☐	Estimates of effect sizes (e.g. Cohen's d, Pearson's r), indicating how they were calculated

Our web collection on statistics for biologists contains articles on many of the points above.

Software and code

Policy information about availability of computer code

Data collection	Stereo Investigator, MicroBrightField; Zeiss AxioScan.Z1 slide scanner (10x/0.45 Plan-Apochromat, Carl Zeiss Microscopy GmbH, Göttingen, Germany); SimoaTM NF-Light Advantage assay Kit (Quanterix, Billerica, MA); Electrochemiluminescence (ECL)-linked immunoassay (Meso Scale Discovery, MSD, Gaithersburg, MD); nanoElute LC coupled online to a timsTOF pro mass spectrometer equipped with a column toaster (Bruker, Germany);Fiji plugin (version 2.0.0-rc-69/1.52p
Data analysis	Prism 9 (GraphPad); Microsoft Excel v.16; Simoa HD-1 Analyzer (Quanterix, Billerica, MA); MSD Discovery Workbench 3.0 (Meso Scale Discovery); Stereo Investigator Version 2018.2.2; Proteome DIA PASEF raw data with DIA-NN Version 1.8.; Perseus (Version 1.6.14)

For manuscripts utilizing custom algorithms or software that are central to the research but not yet described in published literature, software must be made available to editors and reviewers. We strongly encourage code deposition in a community repository (e.g. GitHub). See the Nature Portfolio guidelines for submitting code & software for further information.

Data

Policy information about availability of data

All manuscripts must include a data availability statement. This statement should provide the following information, where applicable:

- Accession codes, unique identifiers, or web links for publicly available datasets
- A description of any restrictions on data availability
- For clinical datasets or third party data, please ensure that the statement adheres to our policy

The data availability statement is included in the manuscript
The mouse mass spectrometry proteomics data have been deposited to the ProteomeXchange Consortium via the PRIDE partner repository with the dataset

identifier PXD032782.

Field-specific reporting

Please select the one below that is the best fit for your research. If you are not sure, read the appropriate sections before making your selection.

☒ Life sciences ☐ Behavioural & social sciences ☐ Ecological, evolutionary & environmental sciences

For a reference copy of the document with all sections, see nature.com/documents/nr-reporting-summary-flat.pdf

Life sciences study design

All studies must disclose on these points even when the disclosure is negative.

Sample size	Pre-determination of the required sample size (mouse number) was done using the statistical power analysis program G*Power and is based on our previous studies. The targeted number of mice per group was 10 for the young and adult groups and 12-15 for aged groups (i.e., short-term treatment, aged and chronic treatments). Slight deviations in numbers are the result of mouse availability from our in-house mouse colony or premature death.
Data exclusions	No data was excluded unless given a reason and clearly stated in the figure legend (outlier test for Supplemental Data 1; insufficient CSF amount for measurements, Fig 4 and Supplemental Fig. 4; imaging processing error, Supplemental Fig. 3).
Replication	All ELISA measurements were done in duplicates as outlined in the method.
Randomization	All samples/mouse numbers had a random code and the experimentator was blinded towards the code. Allocation of mice to groups was randomized for the short-term treatment and for the long-term treatments.
Blinding	The experimentators were blinded towards the code.

Reporting for specific materials, systems and methods

We require information from authors about some types of materials, experimental systems and methods used in many studies. Here, indicate whether each material, system or method listed is relevant to your study. If you are not sure if a list item applies to your research, read the appropriate section before selecting a response.

Materials & experimental systems

n/a	Involved in the study	
☐	☒	Antibodies
☒	☐	Eukaryotic cell lines
☒	☐	Palaeontology and archaeology
☐	☒	Animals and other organisms
☒	☐	Human research participants
☒	☐	Clinical data
☒	☐	Dual use research of concern

Methods

n/a	Involved in the study	
☒	☐	ChIP-seq
☒	☐	Flow cytometry
☒	☐	MRI-based neuroimaging

Antibodies

Antibodies used	CN6 (in house production); anti-Iba1 antibody (Wako Chemicals)
Validation	CN6 antibody has been vaildated in multiple studies such as Eisele, Y. S. et al. Peripherally applied Aβ-containing inoculates induce cerebral β-amyloidosis. Science (New York, N.Y.) 330, 980–982 (2010) or Uhlmann, R. E. et al. Acute targeting of pre-amyloid seeds in transgenic mice reduces Alzheimer-like pathology later in life. Nature Neuroscience 23, 1580–1588 (2020). The specificity of the Iba-1 antibody for staining has also been validated in multiple studies such as Füger, P. et al., Microglia turnover with aging and in a Alzheimer´s model via long-term in vivo single-cell imaging. Nature Neuroscience 20, 1371–1376 (2020)

Animals and other organisms

Policy information about studies involving animals; ARRIVE guidelines recommended for reporting animal research

Laboratory animals	Female and male heterozygous C57BL/6J-TgN(Thy1-APPSw,Thy1-PSEN1*L166P)21 (APPPS1) mice as well as non-transgenic wildtype (WT) mice were used. The age of the animals and numbers are detailed in Figure 1b and Supplementary Fig. 1a. Heterozygous C57BL/6J-TgN(Thy1.2-hAPP751-KM670/671NL)23 (APP23) mice were used as hosts for the in vivo seeding assay. All mice were bred at the Hertie Institute for Clinical Brain Research (Tübingen, Germany) and kept under specific pathogen-free conditions. APP23 mice overexpress the human APP transgene harboring the Swedish double mutation under the neuron-specific Thy1 promoter element. APPPS1 mice carry, in addition to the transgene of APP23 mice, a mutation in PSEN-1, which encodes for presenilin-1, a subunit of

	gamma-secretase. All animal experiments were approved by the local Animal Care and Use Committee and in accordance with the veterinary office regulations of Baden-Wuerttemberg (Germany). All mice were kept under specific pathogen-free conditions with 22 +2°C temperature, 55+10% humidity, a 12 h light/dark cycle (06.00-18.00), and with food and water ad libitum. Commercially available rodent chow (Altromin #1324) was used
Wild animals	The study did not involve wild animals.
Field-collected samples	No field-collection samples were used.
Ethics oversight	The experimental procedures were carried out in accordance with the veterinary office regulations of Baden-Wuerttemberg (Germany) and approved by the local Animal Care and Use Committee (Regierungspräsidium Tübingen, Konrad-Adenauer-Straße 20, 72072 Tübingen).

Note that full information on the approval of the study protocol must also be provided in the manuscript.
